# Protein arginine methylation in viral infection and antiviral immunity

**DOI:** 10.7150/ijbs.89498

**Published:** 2023-10-24

**Authors:** Kai Zheng, Siyu Chen, Zhe Ren, Yifei Wang

**Affiliations:** 1 School of Pharmacy, Shenzhen University Medical School, Shenzhen University, Shenzhen, 518055, China.; 2 Institute of Biomedicine, College of Life Science and Technology, Guangdong Province Key Laboratory of Bioengineering Medicine, Key Laboratory of Innovative Technology Research on Natural Products and Cosmetics Raw Materials, Jinan University, Guangzhou, 510632, China.

**Keywords:** Protein arginine methyltransferase, antiviral immunity, arginine methylation, post-translational modifications, viral infection.

## Abstract

Protein arginine methyltransferase (PRMT)-mediated arginine methylation is an important post-transcriptional modification that regulates various cellular processes including epigenetic gene regulation, genome stability maintenance, RNA metabolism, and stress-responsive signal transduction. The varying substrates and biological functions of arginine methylation in cancer and neurological diseases have been extensively discussed, providing a rationale for targeting PRMTs in clinical applications. An increasing number of studies have demonstrated an interplay between arginine methylation and viral infections. PRMTs have been found to methylate and regulate several host cell proteins and different functional types of viral proteins, such as viral capsids, mRNA exporters, transcription factors, and latency regulators. This modulation affects their activity, subcellular localization, protein-nucleic acid and protein-protein interactions, ultimately impacting their roles in various virus-associated processes. In this review, we discuss the classification, structure, and regulation of PRMTs and their pleiotropic biological functions through the methylation of histones and non-histones. Additionally, we summarize the broad spectrum of PRMT substrates and explore their intricate effects on various viral infection processes and antiviral innate immunity. Thus, comprehending the regulation of arginine methylation provides a critical foundation for understanding the pathogenesis of viral diseases and uncovering opportunities for antiviral therapy.

## Protein Arginine methylation

**Figure 1:**
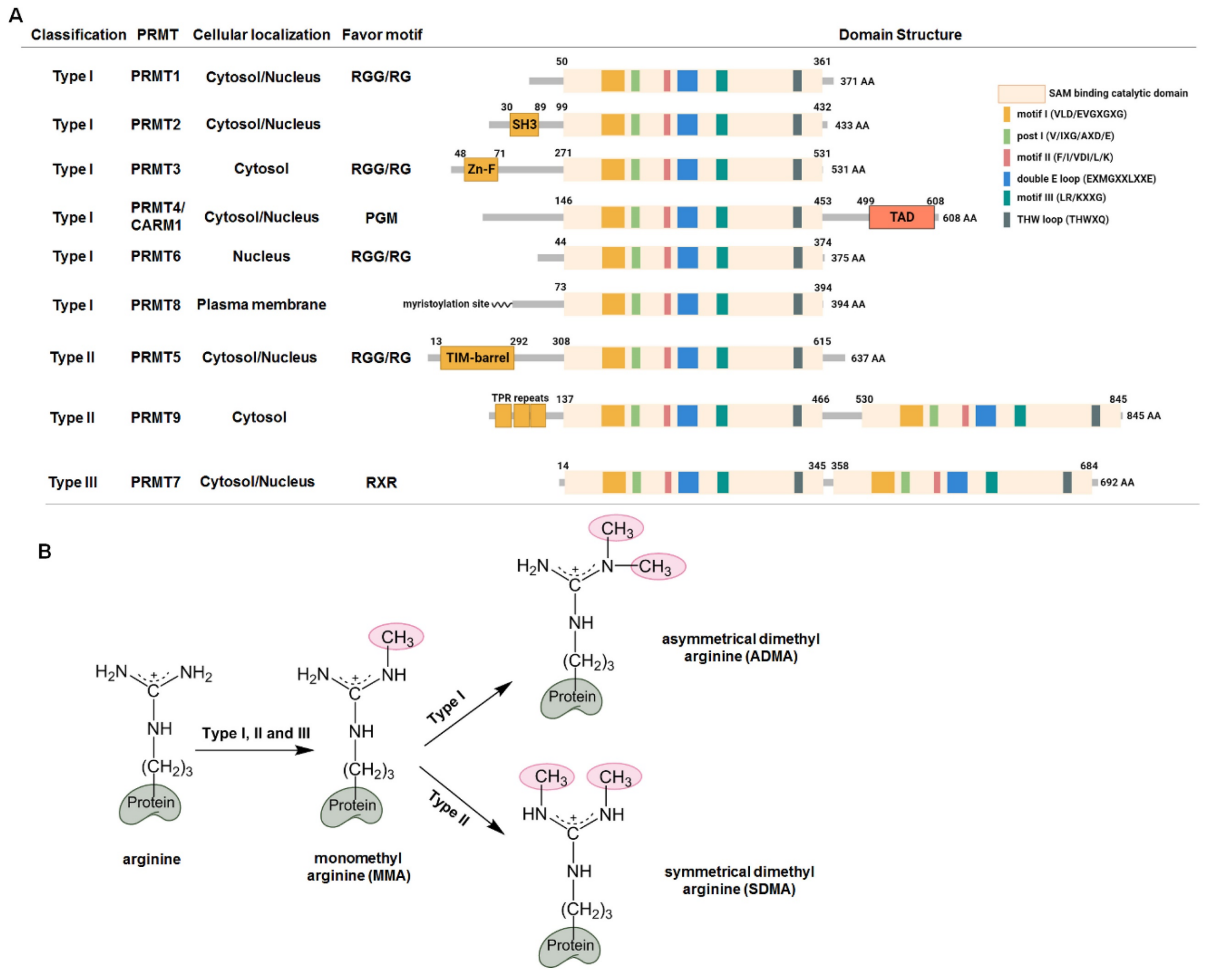
** Arginine methylation and protein arginine methyltransferases (PRMTs). (A)** Classification and structural domains of PRMTs. The S-adenosyl-L-methionine (SAM) binding and catalytic active domain consists of six conserved motifs. The preferences of the arginine motifs for the PRMTs are as follows: PRMT1, PRMT3, PRMT5 and PRMT6 preferentially methylate arginines within the arginine (R)-glycine (G) or RGG motif; PRMT7 has a preference for arginines within the RXR sequences; CARM1 selectively methylates arginines with neighboring proline, glycine and methionine (PGM). SH3, SH3 domain; Zn-F, zinc finger domain; TAD, transactivation domain; TPR, the tetratricopeptide repeat. **(B)** Arginine methylation by PRMTs. Type I, II and III enzymes initially transfer methyl group to the nitrogen atoms of the guanidino group to form monomethyl arginine (MMA), which is further catalyzed by type I enzymes to generate asymmetrical dimethyl arginine (ADMA), or by type II enzymes to form symmetrical dimethyl arginine (SDMA).

The biophysical properties of proteins, including activity, stability, interaction, and localization, are influenced by several well-established posttranslational modifications (PTMs), including acetylation, phosphorylation, sumoylation, and ubiquitination. Arginine methylation is another commonly observed PTM, in which the nitrogen atom of the arginine guanidinium group is modified by methyl groups, resulting in its protonation at physiological pH with a loss of hydrogen bonding capacity and increased hydrophobicity 1. Such modification alters the essential intramolecular and intermolecular interactions (van der Waals forces, hydrogen bonding, and π-π stacking) between proteins and nucleic acids, ultimately affecting the structure and function of target proteins 2,3. Specifically, arginine methylation is catalyzed by protein arginine methyltransferases (PRMTs, called PRMT1-9 in mammals) - often called the 'writers' (Figure 1). This modification can also be reversibly demethylated by arginine demethylases known as Jumonji C domain-containing proteins (JMJDs) - termed the 'erasers' 4,5. Methylarginines are further recognized by proteins containing domains such as Tudor and WD40 domains, as well as plant homeodomain zinc fingers, referred to as the 'readers' 6. Arginine methylation primarily occurs within the arginine-glycine (RG)-, RGG-, or GRG-rich motifs found in numerous proteins, including histones and non-histones 7-10. While histone arginine methylation epigenetically regulates gene expression, arginine methylation of non-histones, such as RNA-binding proteins (RBPs), affects their physical association with nucleic acids, thereby regulating RNA splicing, translation, and cellular signaling in response to stress 11,12. Widely reported proteins with arginine-methylated RG/RGG motifs further expand the regulatory repertoire and potential biological functions of PRMTs 7,11.

The substrate range and biological functions of arginine methylation in cancer, neurological disorders, and inflammatory diseases have been extensively discussed 2,13,14. A myriad of distinct PTMs are deployed by viral infections as connectors in virus-host interactions 15,16. Accumulating evidence illustrates the pleiotropic roles of PRMTs and arginine methylation in diverse cellular and viral infection-associated processes. In this review, we discuss the classification, substrates, structures, and PTM-mediated regulation of PRMTs, as well as their pleiotropic biological functions through the methylation of histones and non-histones. We also summarize the broad and complex effects of arginine methylation on viral infection and antiviral immunity and highlight potential antiviral therapeutic options for the exploration of arginine methylation and PRMT inhibitors.

## Classification, substrates, and structures of PRMTs

Since the identification of the first PRMT (now named PRMT1) by Paik and Kim in 1967 17,18, concomitant with the discovery of arginine-methylated proteins, at least nine PRMT enzymes have been identified in mammals 1. PRMTs transfer the methyl groups from the donor cofactor S-adenosyl-L-methionine (SAM) to the ω-guanidino nitrogen atom of arginine, resulting in the formation of monomethyl arginine (MMA), symmetric dimethyl arginine (SDMA) (each N-terminal guanidino group containing one methyl group), and asymmetric dimethyl arginine (one terminal guanidino group containing two methyl groups) (Figure 1B). Accordingly, PRMTs are classified into three types: Type I enzymes, which asymmetrically dimethylate their substrates, including PRMT1, PRMT2, PRMT3, PRMT4/CARM1 (co-activator-associated arginine methyltransferase 1), PRMT6, and PRMT8; Type II enzymes, which symmetrically dimethylate their substrates, including PRMT5 and PRMT9; and Type III enzymes, which monomethylates substrates, represented solely by PRMT7. Notably, mass spectrometry suggested that PRMT1 and PRMT5 may also catalyze the monomethylation of their substrates 19,20. In addition, PRMT1 and PRMT5 exhibit overlapping substrate specificity, and they can competitively modify the same substrates, effectively substituting for each other to perform similar methyltransferase functions on the same substrate 21. Additionally, the arginine methylation of identical substrates by different PRMTs may serve mutually exclusive functions 1, and only a small number of proteins have been unambiguously assigned to arginine methylation by a single PRMT 22. In structural terms, all PRMTs possess a conserved SAM-binding and catalytic active site composed of seven β-stranded domains. They form two opposing catalytic sites in their head-tail homodimer, except for PRMT7, which functions as a monomer 1. The presence of double E-loops (EXMGXXLXXE) defines the recognition and methylation of substrates, whereas the diverse N-terminal domains make them unique.

### Type I PRMT

Of the nine PRMTs, PRMT1 is the most abundant and potent enzyme, with both cytosolic and nuclear localization accounting for 85% of PRMT activity 23. PRMT1 homodimerizes and selectively methylates RGG/RG motifs, which rely on two critical methionine residues (M48 and M155) that alter these amino acids, resulting in a marked change in substrate specificity and a reduction in catalytic activity 24. Seven different isoforms of PRMT1 with distinct N-termini have been identified by alternative splicing, each with unique activity, substrate preference, and localization 25. Interestingly, inhibition of PRMT1 causes a complementary elevation in PRMT5-mediated SDMA and MMA levels, which may result in opposite outcomes due to substrate scavenging 21. PRMT3 is predominantly cytosolic and has a unique N-terminal zinc finger domain that governs its substrate selectivity and catalytic activity by recruiting binding partners such as DAL-1/4.1B 26,27. In contrast to PRMT1 and PRMT3, PRMT4/CARM1 mainly targets and methylates proline-rich sequences through its unique N-terminal EVH1 domain 28. CARM1 also has a unique C-terminal transactivation domain that serves its transcriptional co-activator function. Both PRMT6 and PRMT8 are expressed in neurons, with PRMT6 predominantly localized in the nucleus, catalyzing arginine methylation of RGG/RG motifs 29,30. Conversely, PRMT8 is anchored to the plasma membrane via N-terminal myristoylation and functions as a tetramer for substrate recognition and methylation 31.

### Type II PRMT

Most of the SDMA formation catalyzed by type II enzymes is attributed to PRMT5, which forms a tight hetero-octameric complex with methylosome protein 50 (MEP50, also known as WD40 repeat-containing protein 77; WDR77) to selectively methylate substrates with GRG sequences 20,32. The PRMT5:MEP50 complex also interacts with several substrate adaptors, such as pICln (chloride nucleotide-sensitive channel 1A), CoRP5 (cooperator of PRMT5), Menin, RioK1 (Rio kinase 1), and Piwi 33. These partners mutually bind exclusively to the N-terminal domain of PRMT5 to determine the substrate choice and catalytic activity. PRMT9 is another type II enzyme with two SAM-binding and catalytic domains (an active site architecture similar to that of PRMT7) and forms a pseudodimer for substrate-binding and activity 34,35. PRMT9 does not methylate arginine within RGG/RG motifs. Furthermore, only one of its substrates, spliceosome-associated protein 145, has been identified, implying that PRMT9 may play a regulatory role in alternative splicing 35,36.

### Type III PRMT

As the only type III enzyme, PRMT7 possesses a more confined substrate-binding cavity. The two conserved double E-loops are critical for its substrate preference, and mutations in these loops can cause PRMT7 to function as a type I or II enzyme 34,37,38. PRMT7 predominantly methylates substrates with an RXR sequence, distinct from the preferences of type I and II enzymes 39. Notably, nonfunctional mutations in the human *PRMT7* gene have been reported, and both homozygous and heterozygous mutations have been linked to the short stature, brachydactyly, intellectual developmental disability, and seizures (SBIDDS) syndrome 40.

## Arginine methylation of histones and non-histones

Similar to other PTM-related kinases, PRMTs methylate the arginine residues of both histone and non-histone proteins, playing essential roles in gene regulation, genome stability maintenance, RNA maturation, and cellular stress-responsive signaling 1-3,41. Importantly, the abnormally high expression of certain PRMTs also indicates their involvement in numerous diseases, such as tumorigenesis, neurodegeneration, and inflammation, as extensively reviewed elsewhere 13,14.

### Histone methylation by PRMTs

**Figure 2:**
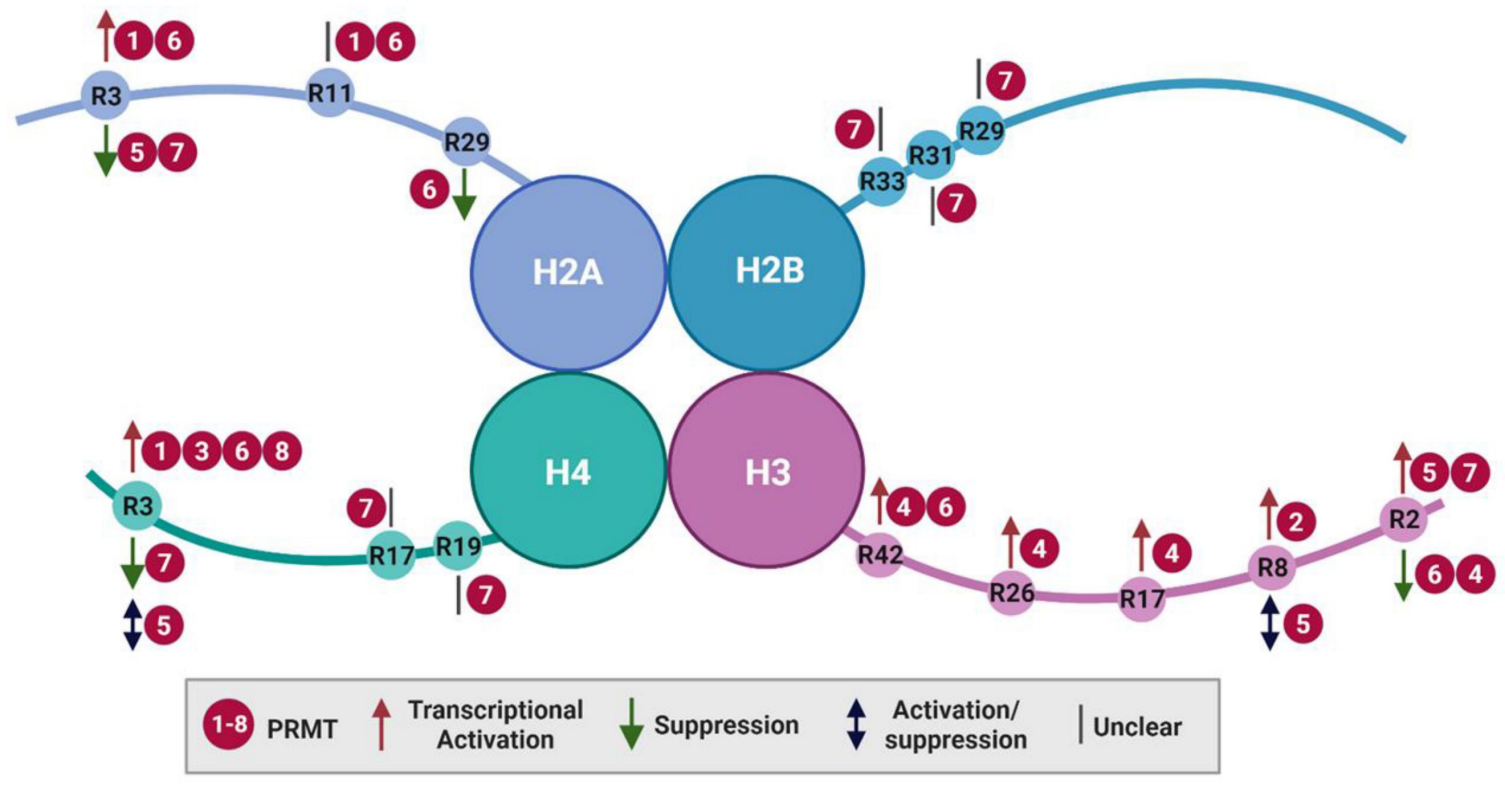
** Histone arginine methylation by PRMTs.** Key arginine (R) residues methylated by PRMTs on histone tails and their consequences for transcriptional regulation are illustrated. PRMT, protein arginine methyltransferases.

Histone arginine methylation is closely linked to transcriptional regulation, DNA damage repair, and chromatin reorganization 42. Although individual PRMTs may have preferences for different core histone substrates (H2A, H2B, H3, and H4), most arginine methylations imply the activation of gene expression (Figure 2 and Table 1). Notably, PRMT9 does not appear to methylate histones, and only a single histone catalytic site has been identified for PRMT2 and PRMT3 that maintains the expression of specific target genes. Alternatively, the dynamic crosstalk between different histone modifications, such as acetylation and methylation, serves as context-dependent histone markers that influence either active or repressive gene expression.

#### H2A methylation

Arginine 3 of H2A (H2AR3) can be methylated by PRMT1, PRMT5, PRMT6, and PRMT7. PRMT1 and PRMT6 catalyze asymmetric demethylation (H2AR3me2a), associated with transcriptional activation 43-45, whereas PRMT5-mediated methylation (H2AR3me1/me2s) 46 and PRMT7-mediated symmetric demethylation (H2AR3me2s) 47 represent gene silencing. In addition, PRMT1 and PRMT6 can methylate H2A at R11 (H2AR11me2a), the biological consequences of which remain unclear 48. Furthermore, PRMT6 catalyzes the methylation of R29 (H2AR29me2a), a mark associated with transcriptional repression 43,48.

#### H2B methylation

To date, only PRMT7 has been reported to monomethylate H2B at R29, R31, and R33. The functional implications of these modifications for gene expression are yet to be determined 39. The presence of an RXR motif (′29-RKRSR-33′) in the N-terminus of H2B is indispensable for PRMT7 methylation, with minor changes in the sequence dramatically affecting PRMT7 activity. This may explain its narrow substrate specificity 49.

#### H3 methylation

Arginine methylation of H3 occurs at residues R2, R8, R17, R26 and R42. In most cases, H3 methylation by PRMTs exerts transcriptional activation, with exceptions for PRMT6-mediated H3R2me2a and PRMT5-mediated H3R8me2s 50-52. For instance, CARM1 catalyzes H3R17me2a, H3R26me2a, and H3R42me2a 53-55. PRMT6 catalyzes H3R42me2a to stimulate gene transcription 56. Additionally, CARM1 and PRMT5-7 methylate H3R2 with different outcomes. Initially, the methylation of H3R2me2a was identified as an enzyme activity mediated by CARM1 *in vitro* 53. Subsequent research revealed that PRMT6 is the primary catalyst responsible for this methylation, which exerts its effects by inhibiting gene expression through its interaction with H3 lysine 4 trimethylation (H3K4me3) and interfering with H3K4me3 reader binding 45,56. In contrast, H3R2 modification (H3R2me1/me2s) by PRMT5 is associated with transcriptional activation 57,58, which activates and recruits WD-40 repeat-containing protein 5 (WDR5) to promote H3K4me3 and gene transcription 59,60. One possible explanation is that WDR5 has high binding affinity for H3R2me2s, which is obstructed by H3R2me2a 61,62. Furthermore, symmetric demethylation of H3R2 is catalyzed by PRMT7 and is detected in euchromatic transcriptional activation regions 37,57. H3R8me2a is mainly generated by PRMT2 and accumulates at the promoters and enhancers of transcriptionally active target genes 63. In contrast, PRMT5-mediated H3R8me2s mainly serves as a repressive mark 57,58. However, in some cases, PRMT5 acts through H3R8me2s to upregulate specific genes in cancer, such as those related to eukaryotic initiation factor 4E, fibroblast growth factor receptor 3, and androgen receptor 51,52.

### H4 methylation

H4 is mainly methylated at R3 by PRMT1, PRMT3, and PRMT5-8, which exert either positive or negative effects on gene transcription. For example, PRMT1 catalyzes H4R3me2a to epigenetically regulate the epithelial-to-mesenchymal transition process 64 or to recruit the chromatin remodeling component SMARCA4 to upregulate genes involved in epidermal growth factor receptor signaling in colorectal cancer 65. PRMT8 exhibits the highest homology with PRMT1 and demonstrates similar substrate activity in mediating H4R3 modifications 31. In addition, PRMT3- and PRMT6-mediated H4R3me2a promote the transcriptional activation of certain genes 66,67. In contrast, PRMT5-mediated H4R3me2s have complex and context-dependent functions in gene expression via altered PRMT5 adaptor proteins. PRMT5 methylation of H4R3me2s can inhibit gene expression via DNA methylation or histone ubiquitination 68. Conversely, similar to H3R8me2s, it can upregulate genes or be associated with H4K5 acetylation, serving as an active marker of expression 58,69. Additionally, H4R3me2s modification by PRMT7 is belived to suppress gene expression by binding to gene promoters. This is associated with increased H4R3me2s levels and reduced H3K4me3, H3, and H4 acetylation 70,71. Interestingly, PRMT7 also methylates H4 R17 and R19. H4R17me1 enhances PRMT5-mediated H4R3me2s, which, in turn, inhibits subsequent H3K4 methylation and H3K9 acetylation 72,73. Therefore, different histone modifications, such as methylation and acetylation, cooperatively influence gene expression.

### Non-histone methylation by PRMTs

In addition to histones, PRMTs target numerous substrates and interacting partners involved in transcriptional regulation, DNA repair, RNA processing, and cellular metabolism 2,13,74. Over 4,000 proteins with arginine methylation at phosphosites have been documented 75. These non-histone substrates include transcription factors, RNA splicing factors, binding proteins, and cellular signal transducers 76. For example, RBPs are essential for RNA metabolism, such as splicing, maturation, translation. PRMTs recognize and methylate arginine residues within the RG/RGG-rich motifs of RBPs. This modification interferes with protein-protein and protein-nucleic acid interactions, thereby affecting RNA-associated processes 77. In addition, proteome-wide analysis shows that numerous extensively arginine-methylated proteins are enriched in RNA splicing 20. For example, PRMT5 methylates Sm proteins, a type of RBP, promoting the formation of spliceosomal small nuclear ribonuclear protein complexes 78. It also methylates splicing factors to facilitate precise alternative splicing 79. Additionally, PRMTs methylate proteins from multiple layers of the DNA damage response, such as BRCA1 (Breast cancer susceptibility gene 1), 53BP1 (p53 binding protein 1), and MRE11 (meiotic recombination 11), regulating their transcription, stability, activity, subcellular location, and protein or nucleic acid interactions to adapt to genotoxic stimuli 80-82. Furthermore, PRMT-mediated arginine methylation has been implicated in several diseases, including cancer, neurodegenerative disorders, and inflammatory diseases, because of the wide range of functional substrates and biological processes involved 13,14. Extensive research has been conducted on the potential regulatory functions of PRMTs in these diseases, offering promising therapeutic avenues, the details of which are not discussed herein.

## Regulation of PRMT activity by PTMs

Accumulating evidence has revealed that PRMT activity and substrate specificity are regulated by various PTMs, including phosphorylation, ubiquitination, methylation, and O-GlcNAcylation. These PTMs play a pivotal role in shaping the diverse functions of PRMTs in various cellular processes. However, of the nine PRMTs, only the regulation of PRMT1, CARM1, and PRMT5 has been documented (Table 2).

### Regulation of PRMT1

**Figure 3:**
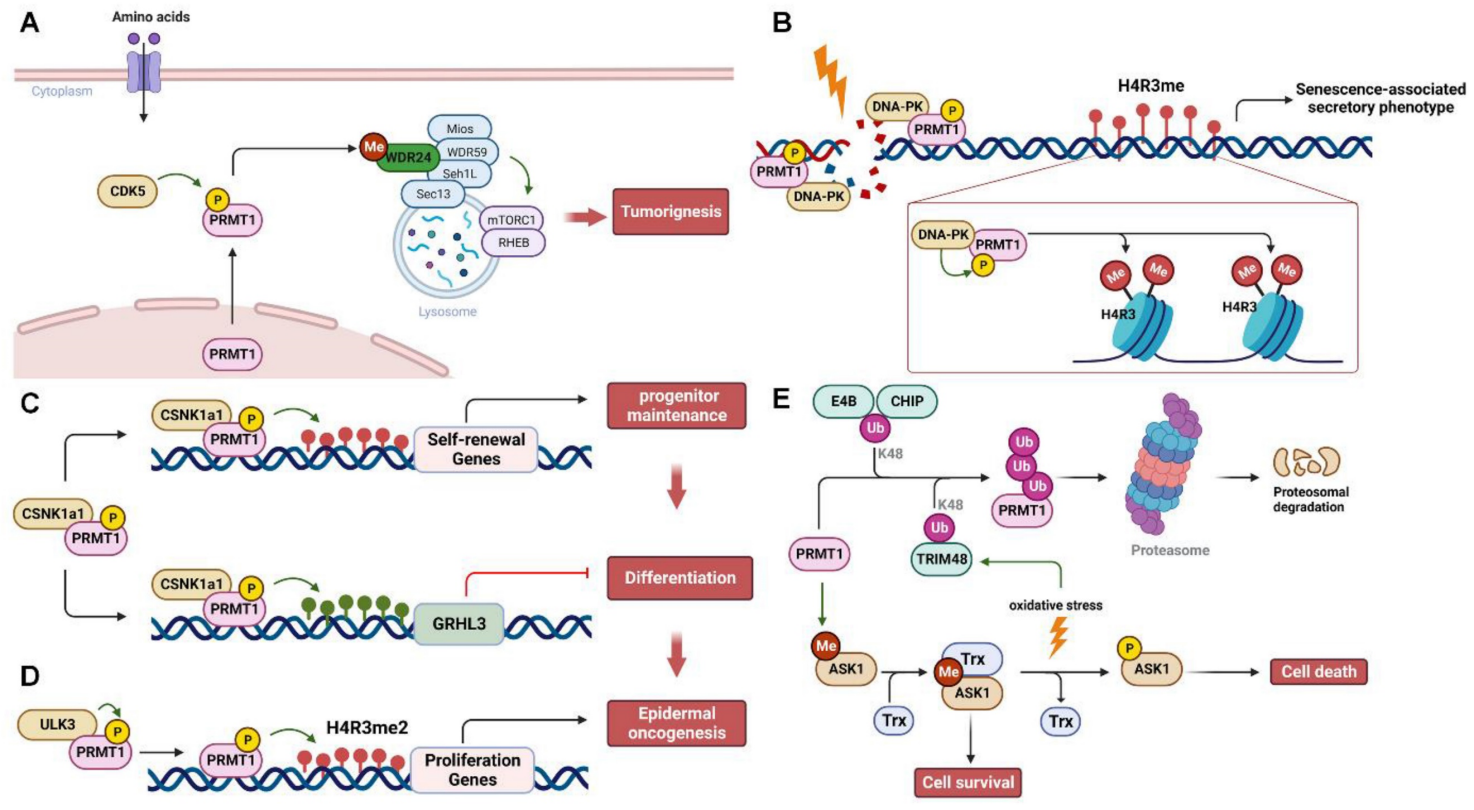
** Regulation of PRMT1 by post-translational modifications. (A)** PRMT1 is phosphorylated and activated by CDK5 to methylate WDR24, resulting in mTORC1 activation and tumorigenesis upon amino acid stimulation. **(B)** DNA damage caused by cisplatin triggers the binding of DNA-PK to PRMT1, which in turn phosphorylates PRMT1 and promotes the H4R3 methylation, leading to the senescence-associated secretory phenotype. **(C and D)** PRMT1 is phosphorylated by CSNK1a1 or ULK3 to epigenetically regulate the expression of genes involved in the epidermal self-renewal and oncogenesis via histone methylation. **(E)** PRMT1 is either ubiquitinated by E4B, CHIP or TRIM48 for proteasomal degradation, or deubiquitinated by USP11 for DNA damage repair. P, phosphorylation; Me, methylation; Ub, ubiquitination. ASK1, apoptosis signal-regulating kinase 1; CDK5, cyclin-dependent kinase 5; CHIP, carboxy-terminus of Hsc70 interacting protein; CSNK1a1, casein kinase 1α; DNA-PK, DNA-dependent protein kinase; GRHL3, grainyhead like transcription factor 3; MRE11, meiotic recombination 11; PRMT1, protein arginine methyltransferase 1; Trx, thioredoxin; WDR24, WD-40 repeat-containing protein 24; ULK3, Unc51-likekinase 3; USP11, ubiquitin specific peptidase 11.

PRMT1 activity and stability are subject to regulation through phosphorylation and ubiquitination (Figure 3). Under various stress stimuli, different upstream kinases phosphorylate PRMT1 and regulate its translocation and methyltransferase activity to control specific substrate methylation and signaling pathways. For instance, upon amino acid stimulation, cyclin-dependent kinase 5 (CDK5) phosphorylates PRMT1 at S307 to promote its transportation to lysosomes in the cytoplasm for the methylation of WDR24 and subsequently activates the mTORC1 pathway, leading to tumor growth 83 (Figure 3A). In response to genotoxic stress induced by cisplatin, DNA-dependent protein kinase (DNA-PK) phosphorylates and recruits PRMT1 to promote H4R3me2s methylation, resulting in the senescence-associated secretory phenotype 84 (Figure 3B). During the self-renewing process of the epidermis, casein kinase 1α (CSNK1a1) phosphorylates PRMT1 at S248/T285/S289, regulating its chromatin localization and influencing the expression of target genes involved in maintaining self-renewal 85. Additionally, PRMT1 represses the expression of grainyhead-like transcription factor 3 (GRHL3), a pro-differentiation transcriptional activator, ultimately inhibiting the terminal differentiation of progenitors 85 (Figure 3C). In contrast, Unc51-like kinase 3 (ULK3) interacts with and phosphorylates PRMT1 to enhance its methyltransferase activity and promote chromatin recruitment. This, in turn, increases H4R3 methylation and cell proliferation, leading to the tumorigenic differentiation of epidermal keratinocytes, ultimately facilitating epidermal oncogenesis 86 (Figure 3D). Notably, PRMT1 phosphorylation at Y291 impairs its ability to methylate substrates, resulting in altered substrate specificity and protein interactions 87. In addition to phosphorylation, two E3 ubiquitin ligases, CHIP (carboxy-terminus of Hsc70 interacting protein) and E4B (ubiquitination factor E4B), recognize and ubiquitinate PRMT1 for proteasomal degradation 88. Furthermore, Tripartite motif 48 (TRIM48) is another E3 ubiquitin ligase that promotes K48-linked polyubiquitination of PRMT1, leading to its proteasomal degradation. This process activates apoptosis signal-regulating kinase 1 (ASK1) and, under oxidative stress, induces cell death 89. PRMT1-mediated methylation promotes the binding of ASK1 to its negative regulator thioredoxin (Trx), thereby inactivating ASK1 to ensure cell survival. In contrast, PRMT1 ubiquitination by TRIM48 prevents the ASK1-Trx interaction and facilitates ASK1 autophosphorylation, thereby suppressing cancer cell growth (Figure 3E). Additionally, USP11 deubiquitylates PRMT1 to regulate its interaction with the DNA repair protein MRE11, thereby controlling DNA damage repair 90.

### Regulation of CARM1

**Figure 4:**
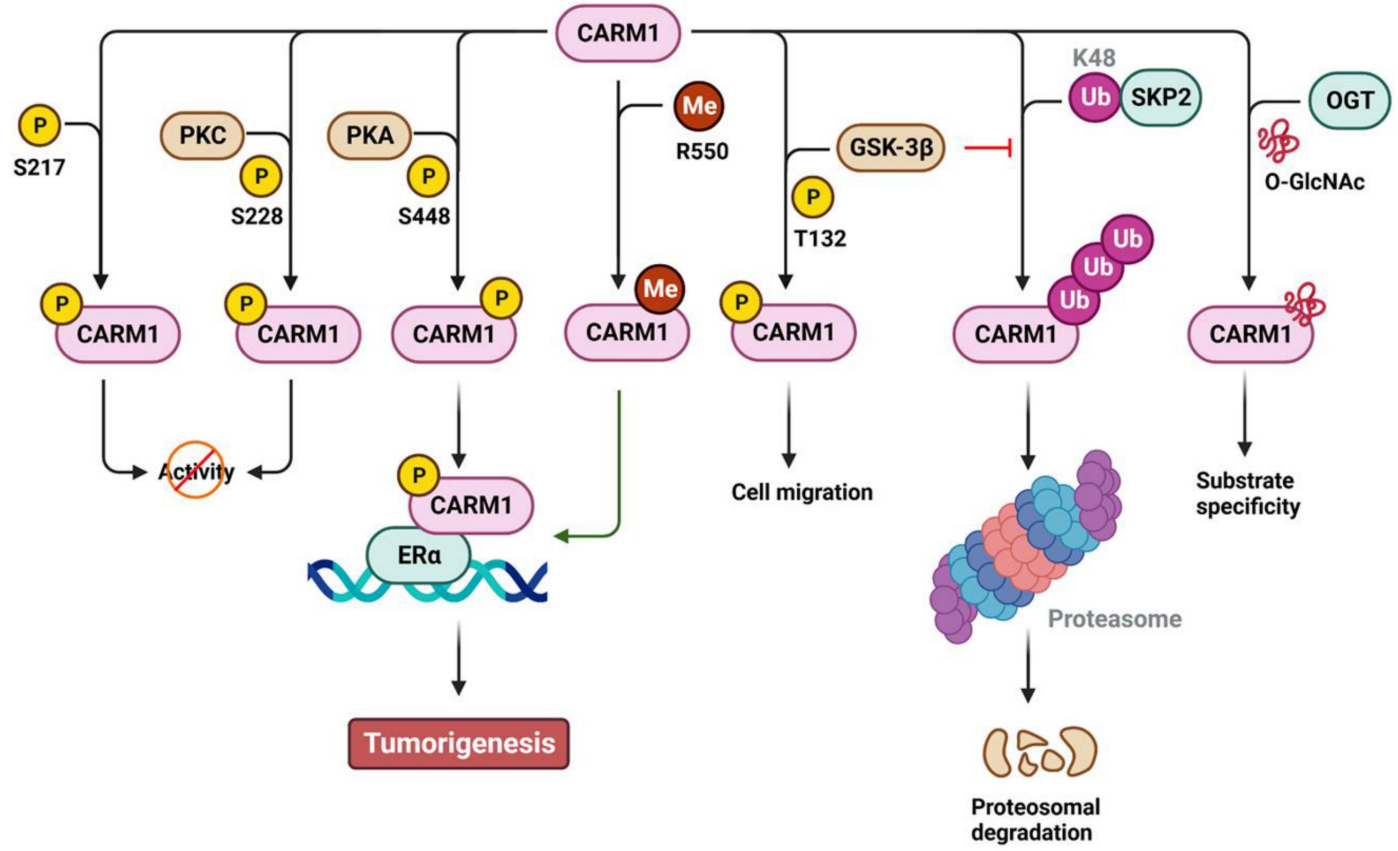
** Regulation of CARM1 by post-translational modifications.** The activity, stability, substrate specificity and the biological consequences of CARM1 are regulated by phosphorylation (P), methylation (Me), ubiquitination (Ub) and O-GlcNAcylation (GlcNAc). The amino acid residues modified by PTMs are also shown. K, lysine; R, arginine; S, serine; T, threonine; Y, tyrosine. CARM1, coactivator-associated arginine methyltransferase 1; ERα, estrogen receptor α; GSK-3β, glycogen synthase kinase 3β; PKA, protein kinase A; PKC, protein kinase C; OGT, O-GlcNAc transferase; SKP2, S-phase kinase-associated protein 2.

Similar to PRMT1, CARM1 can be phosphorylated to regulate its activity, stability, and co-activator function in a positive or negative manner (Figure 4). Specifically, phosphorylation of CARM1 at S217 by unknown kinase(s) blocks the binding of SAM to the catalytic site, whereas phosphorylation at S228, possibly by protein kinase C (PKC), disrupts CARM1's dimerization, ultimately restricting its methyltransferase activity during mitosis or dendritic maturation 91-93. Phosphorylation of S448 by protein kinase A (PKA) promotes CARM1 binding to the estrogen receptor α (ERα), acting as a cofactor for ERα-responsive gene transcription and breast carcinogenesis 94. In addition, phosphorylation of T132 by glycogen synthase kinase 3β (GSK-3β) prevents its ubiquitination and subsequent proteasomal degradation, thereby restoring lung epithelial cell migration that has been impaired by oxidative stress 95. Specifically, the stability of CARM1 is controlled by polyubiquitination at K471 mediated by SCF E3 ubiquitin ligase S-phase kinase-associated protein 2 (SKP2) 96. Under nutrient-rich conditions, SKP2 actively catalyzes the ubiquitination and degradation of CARM1 in the nucleus. Conversely, under nutrient-starved conditions, SKP2 expression is transcriptionally suppressed by the phosphorylation of forkhead box O3a (FOXO3a) by AMP-activated protein kinase. This leads to increased levels of CARM1-mediated H3R17me2 and induces autophagy. In addition to phosphorylation, approximately 50% of CARM1 is modified by O-GlcNAcylation at S595, S598, T601, and T603. This modification plays a role in determining CARM1's substrate specificity 97,98 and implicates O-GlcNAcylated CARM1 as a sensor of metabolic status and cellular stress. Moreover, CARM1 is automethylated at R550 (R551 in mouse) in exon 15. Such automethylation does not affect its enzymatic activity but rather alters ERα transcription and alternative mRNA splicing 99. Alternative splicing of CARM1 mRNA generates two isoforms: full-length CARM1 (automethylated) and exon 15 deleted CARM1ΔE15 (automethylation-defective). These isoforms display differential distribution in stromal and epithelial cells, as well as different substrate methylation activity 100. In addition, the stromal-predominant CARM1ΔE15 isoform loses its co-activator function in regulating ERα transcription, suggesting distinct roles for ERα in epithelial and stromal tissues 100.

### Regulation of PRMT5

**Figure 5:**
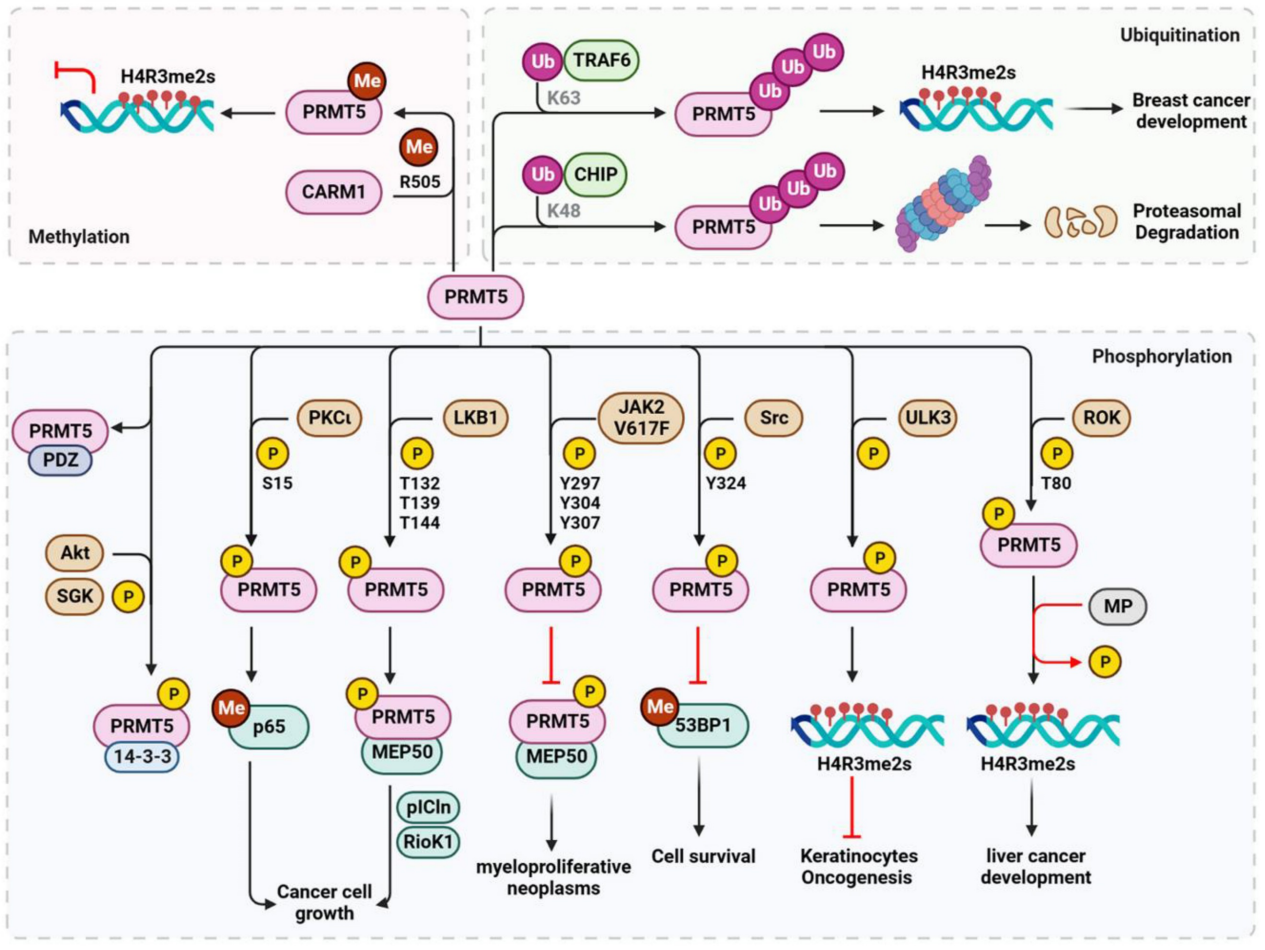
** Regulation of PRMT5 by post-translational modifications.** Stress-responsive kinases phosphorylate PRMT5 at various residues to modulate PRMT5 activity, stability and substrate specificity and their biological consequences, particularly in cancer. PRMT5 is also regulated by K48-linked ubiquitination for proteasomal degradation or K63-linked ubiquitination for histone methylation and tumorigenesis. PRMT5 is further methylated by CARM1 to inhibit gene expression via histone H4R3 methylation. P, phosphorylation; Me, methylation; Ub, ubiquitination. The amino acid residues modified by PTMs are also shown. K, lysine; R, arginine; S, serine; T, threonine; Y, tyrosine. CARM1, coactivator-associated arginine methyltransferase 1; CHIP, carboxy-terminus of Hsc70 interacting protein; JAK2V617F, constitutively active Janus kinase 2 mutant; LKB1, liver kinase B1; MP, myosin phosphatase; MEP50, methylosome protein 50; ROK, RhoA activating kinase; RioK1, Rio kinase 1; PDZ, proteins containing PDZ domain; pICln, chloride nucleotide-sensitive channel 1A; PKCι, Protein kinase C iota; PRMT5, protein arginine methyltransferase 5; SGK, serum and glucocorticoid inducible protein kinase; Src, Src kinase; TRAF6, TNF receptor associated factor 6; ULK3, Unc51-likekinase 3; 14-3-3, 14-3-3 protein; 53BP1, p53-binding protein 1.

Several upstream kinases regulate the phosphorylation of PRMT5 to achieve specific biological functions in response to various stresses, particularly during carcinogenesis (Figure 5). For instance, PKCι phosphorylates PRMT5 at S15 to enhance its methyltransferase activity upon interleukin-1β stimulation. This phosphorylation activates nuclear factor kappa B (NF-κB) through methylation of the p65 subunit at R30, ultimately promoting the growth of colorectal cancer cells 101. In ERα-positive breast cancer, liver kinase B1 (LKB1) interacts with and phosphorylates PRMT5 at T132, T139, and T144 within the nucleus. This phosphorylation event enhances its enzymatic activity and its binding to major co-activators such as MEP50, substrate partner pICln, and RioK1. These interactions determine PRMT5's enzymatic activity toward Sm proteins and histones 102. PRMT5 can be phosphorylated at Y297, Y304, and Y307, inhibiting its activity by Janus kinase 2 (JAK2) mutant JAK2V617F, a constitutively active form of JAK2. This disturbed interaction between PRMT5 and MEP50 serves as a plausible molecular mechanism for myeloproliferative neoplasms 103. Furthermore, in response to normal physiological DNA damage, Src kinase phosphorylates PRMT5 at Y324, blocking its binding to SAM and impairing its methylation of 53BP1, consequently affecting non-homologous end-joining repair and triggering apoptotic cell death 82. PRMT5-mediated histone H4R3me2s methylation is also regulated by phosphorylation. For instance, ULK3 phosphorylates PRMT5, in addition to PRMT1, to enhance its recruitment to chromatin and H4R3me2s methylation, thereby preventing oncogenesis and tumorigenic differentiation of keratinocytes 86. In contrast, RhoA activating kinase (ROK) phosphorylates PRMT5 at T80 enhancing its activity on H4R3me2s and activating proto-oncogenes, contributing to tumor formation in hepatocellular carcinoma. This effect is counteracted by myosin phosphatase (MP)-mediated dephosphorylation at T80 104. Alternatively, in tumor cells, MP is phosphorylated at T850 by ROK, and its diphosphatase activity on PRMT5 is inhibited, leading to the hyperphosphorylation of PRMT5 and increased H4R3me2s. Furthermore, the subcellular location and substrate specificity of PRMT5 are determined by C-terminal phosphorylation. Phosphorylation of T364 by Akt or SGK results in plasma membrane association and binding of PRMT5 to 14-3-3 proteins. Conversely, unphosphorylated PRMT5 binds to proteins containing PDZ domains 105. This phosphorylation event acts as a PDZ/14-3-3 interaction switch, and further studies are required to explore its biological significance.

In addition to phosphorylation, the ubiquitination of PRMT5 affects its stability and methyltransferase activity. The E3 ubiquitin ligase CHIP catalyzes K48-linked polyubiquitination of PRMT5 and its subsequent proteasomal degradation to adapt to cellular stress in prostate cancer cells 106. In contrast, TNF receptor-associated factor 6 (TRAF6), another E3 ubiquitin ligase, promotes K63-linked polyubiquitination of PRMT5, enhancing its methyltransferase activity on histone H4R3 and breast cancer cell proliferation 107. Interestingly, PRMT5 activity is also regulated by methylation. However, unlike CARM1, which requires auto-arginine methylation 99,100, PRMT5 is methylated by CARM1 at R505 108. Such methylation enhances methyltransferase activity and histone H4R3me2s enrichment at the promoter of the γ-globin gene or other genes, ultimately resulting in silenced gene expression. This indicates a critical role for PRMT5 methylation in the pathogenesis of hemoglobinopathies and cancer.

### Regulation of other PRMTs

In addition to the aforementioned PRMTs, phosphorylation plays a role in regulating the activities of PRMT3 and PRMT6, each with different biological functions. PRMT3's catalytic activity and substrate-binding, particularly RPS2, require phosphorylation at Y87, the inhibition of which results in RNA maturation defects 109. In the case of PRMT6, phosphorylation at S11 and T21 by casein kinase 2α (CK2α) inhibits its ubiquitination and proteasomal degradation and increases its binding and subsequent methylation of ribosomal chromatin condensation 1 (RCC1), facilitating RCC1 chromatin recruitment. This, in turn, promotes abnormal proliferation, radiation resistance, and tumorigenicity of glioblastoma stem cells 110. Moreover, similar to arginine methyltransferases, the activities of PRMT6, PRMT7, and PRMT8 undergo automethylation, similar to CARM1. For instance, automethylation of PRMT6 at R35 is essential for its stability and antiretroviral activity 30,111. In contrast, PRMT8 automethylation at R58 and R73 inhibits its enzymatic activity by blocking the binding of SAM to the catalytic site 112,113. However, the biological significance of PRMT7 automethylation is intricate, as several amino acids, including R32, R177, R363, R378, R387, R458, and R531, undergo automethylation 114. Notably, automethylation at R531 increases H4R3me1 levels in genes involved in the epithelial-mesenchymal transition, thereby promoting the progression and metastasis of breast cancer cells 115. Additionally, automethylation at R32 relieves the suppressive function of PRMT7 on the activation of mitochondrial antiviral signaling protein (MAVS) and subsequent antiviral innate immunity (more details are discussed below) 114. Future studies should investigate whether other PRMTs, such as PRMT1, PRMT2, and PRMT3, also undergo automethylation to alter their activity and substrate specificity.

## PRMTs regulate viral infection

PRMTs have emerged as critical regulators of viral infections. PRMT-mediated arginine methylation of special host cellular proteins is involved in different steps of the viral life cycle and antiviral innate immune response. For example, arginine methylation controls the diverse proviral and antiviral functions of Ras GTPase-activating protein-binding protein 1 (G3BP1) 116-118. Alternatively, PRMTs methylate different viral proteins containing RG/RGG-like domains to regulate their location, stability, activity, and interaction with proteins or nucleic acids. Consequently, these modifications influence viral replication, transcription, particle release, and the latency reactivation switch (**Table 3**).

### PRMTs in dsDNA virus infection

#### Herpesviridae

**Figure 6:**
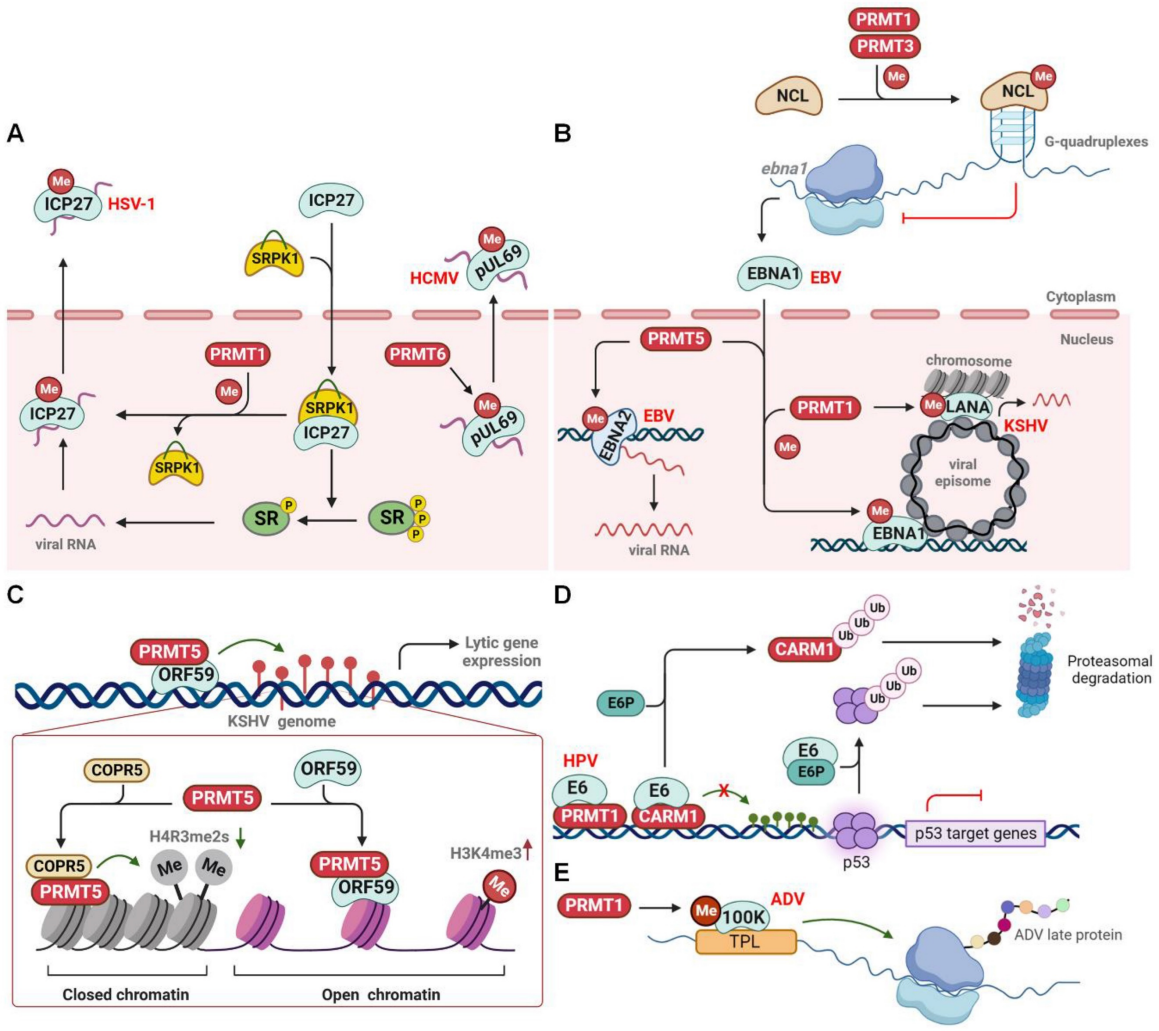
** PRMTs regulate DNA virus infection. (A)** PRMTs regulate the nuclear-cytoplasmic shuttling of viral proteins. **(B)** PRMTs methylate EBV EBNA1 and EBNA2 to regulate viral gene expression, or methylate NCL to promote its binding to G-quadruplexes within EBNA1 mRNA, thereby inhibiting EBNA1 production to prevent immune activation. PRMT1 also methylates KSHV LANA to modulate viral latent reactivation. **(C)** KSHV ORF59 interacts with and suppresses PRMT5-mediated H4R3 methylation to promote viral reactivation. **(D)** HPV E6 inhibits PRMTs-mediated p53-responsive gene expression and triggers CARM1 proteasomal degradation. **(E)** PRMT1 methylates ADV L4-100K to ensure viral late protein synthesis and viral yield. CARM1, coactivator-associated arginine methyltransferase 1; COPR5, cooperator of protein arginine methyltransferase 5; EBV, Epstein-Barr virus; E6P, E6-associated protein; HCMV, human cytomegalovirus; HPV, human papillomavirus; HSV, herpes simplex virus type 1; KSHV, kaposi sarcoma-associated herpesvirus; NCL, nucleolin; SRPK1, serine-arginine protein kinase 1; PRMT, protein arginine methyltransferase; SR, splicing factors; TPL, tripartite leader sequence; Me, methylation; P, phosphorylation; Ub, ubiquitination.

Herpes simplex virus 1 (HSV-1) is a neurotropic, double-stranded (ds) DNA-enveloped virus belonging to the alpha-herpesvirus subfamily. HSV-1 typically infects the body through the mucous membranes, skin, and nerve tissues, causing associated lesions and establishing latency in sensory neurons. The immediate-early protein ICP27 is a multifunctional viral protein that regulates viral replication and promotes host and viral alternative splicing 119. ICP27 induces the nuclear accumulation of serine-arginine protein kinase 1 (SRPK1) and inhibits its phosphatase activity, leading to the hypophosphorylation of splicing factors (SR proteins). This ultimately results in the failure of host spliceosome assembly, facilitating viral transcription and replication (Figure 6A) 16,120. Subsequently, ICP27 disperses from the transcription site and acts as an mRNA export factor/chaperone. It supports the nuclear export of viral RNA, a process that requires PRMT1-mediated arginine methylation at residues R138, R148, and R150 within the RGG motif 121. Notably, PRMT1-mediated methylation also significantly impairs ICP27's interaction with SRPK1 and hinders its nuclear translocation 122,123. Inhibition of the RGG motif methylation by a PRMT1 inhibitor or an arginine substitution causes hypomethylation of ICP27 and dramatically reduces its RNA-binding ability. Consequently, hypomethylated ICP27 forms nuclear foci-like structures or is inappropriately transported to the cytoplasm, resulting in unsuccessful viral replication 121,124. Therefore, PRMT1-mediated arginine methylation of ICP27 acts as a switch to turn off the viral and host genes. In addition to ICP27, the viral protein pUL69 is an ICP27 homologue and multifunctional RNA export factor encoded by the beta-herpesvirus human cytomegalovirus (HCMV). Similarly, pUL69 controls HCMV gene expression and mediates the nuclear export of viral mRNAs 125. Arginine methylation at R22/23 and R25/26 within the N-terminal RG motif is crucial for pUL69-mediated mRNA export and viral replication 126. Although PRMT2 and PRMT6 co-localize with pUL69 within the nucleus, only PRMT6 methylates pUL69 to promote its dissociation from RNA and enhance its interaction with the cellular export factor UAP56 and viral RNA for cytoplasmic transportation (Figure 6A).

Furthermore, infection with Epstein-Barr virus (EBV), a gamma herpes virus, enhances the expression of PRMT1, PRMT5, and CARM1 127. Epstein-Barr nuclear antigen 1 (EBNA1) plays a crucial role in latent EBV infection by initiating viral DNA replication from the latent origin, activating the transcription of latency genes, and segregating episomal viral genomes, all of which require a 325-376 RG-rich region 128. Notably, both PRMT1 and PRMT5 methylate the 325-376 region of EBNA1, regulating its nuclear localization and potential gene transcription (Figure 6B) 129. PRMT5 also methylates another oncoprotein, EBNA2, within the 339-354 RG domain to ensure its active interaction with other viral promoters and DNA-bound transcription factors, thereby enhancing EBNA2 transcriptional activity and viral transcription 130. In contrast, PRMT1 and PRMT3 methylate the host protein nucleolin (NCL) at the C-terminal RGG motif, which promotes the direct binding of NCL to the G-quadruplexes formed in EBNA1 mRNA. This NCL-mediated translational inhibition of EBNA1 ultimately results in the reduced production of EBNA1-derived antigenic peptides and immune evasion of EBV (Figure 6B) 131.

Kaposi sarcoma-associated herpesvirus (KSHV), another gammaherpesvirus, features a latency-associated nuclear antigen (LANA) that is a functional homologue of EBV EBNA1. LANA plays an essential role in establishing and maintaining viral latency. Similar to EBNA1, LANA is methylated at R20 by PRMT1, which affects the transcription of viral gene targets, partly through modulation of the histone binding of LANA (Figure 6B) 132. Interestingly, during lytic KSHV infection, the early expressed viral protein ORF59, which acts as a processivity factor for lytic DNA replication, interacts with PRMT5 and hinders its interaction with the adaptor protein COPR5. This leads to the formation of open chromatin, characterized by the loss of repressive H4R3me2s marks and the enhancement of activating H3K4me3 marks, which ultimately facilitates lytic gene transcription and viral replication (Figure 6C) 133. Taken together, these results suggest that PRMT-mediated arginine methylation regulates the herpes virus lytic latent infection switch and latency maintenance.

#### Papillomaviridae

Human papillomavirus (HPV) is a small, non-enveloped dsDNA virus that causes cervical cancer and other neoplasms in persistent genital and oropharyngeal infections. The early viral protein E6 is critical for transforming and immortalizing infected cells 134. One well-documented oncogenic mechanism involves E6, with the assistance of E6-associated protein (E6P), promoting the proteasomal degradation of the tumor suppressor p53, ultimately contributing to HPV-induced cervical cancer 135. In addition, E6 interacts with and suppresses the methyltransferase activity of PRMT1 and CARM1, leading to reduced histone methylation of p53-responsive promoters and, ultimately, the loss of p53-mediated gene transcription (Figure 6D) 136. Alternatively, E6 triggers the proteasomal degradation of CARM1 in an E6P-dependent manner 137. This strongly suggests that E6 proteins from different HPVs (high- or low-risk) implement diverse strategies to interfere with p53 function and induce abnormal cell proliferation.

#### Adenoviridae

During productive adenovirus infection, the late region 4 (L4) 100-kDa protein (100 K), the first viral late protein, selectively inhibits host cellular protein synthesis and induces the translation of viral late mRNAs to initiate the late phase of viral infection 138. In addition, 100 K interacts with the capsid component of hexon monomers to assist in trimerization, nuclear export, and capsid assembly 139. The C-terminal region of the 100 K protein contains a conserved RGG domain, along with several RG and RXR motifs. These elements undergo PTMs to spatiotemporally regulate the function of 100 K 140. Similar to HSV-1 ICP27, 100 K is methylated and interacts with PRMT1 methylase through its C-terminal RGG domain. This results in nuclear accumulation of 100 K and subsequent 100 K-mediated shutdown of host protein synthesis 141. PRMT1-mediated methylation also facilitates viral late protein production by modulating the binding of 100 K to the tripartite leader sequence (TPL), a conserved 200-nucleotide unit in the 5′-noncoding region of viral late mRNAs. It also regulates hexon biogenesis and viral genome capsidation, which contributes to efficient virus yield (Figure 6E) 142,143.

### PRMTs in dsRNA virus infection

#### Birnaviridae

**Figure 7:**
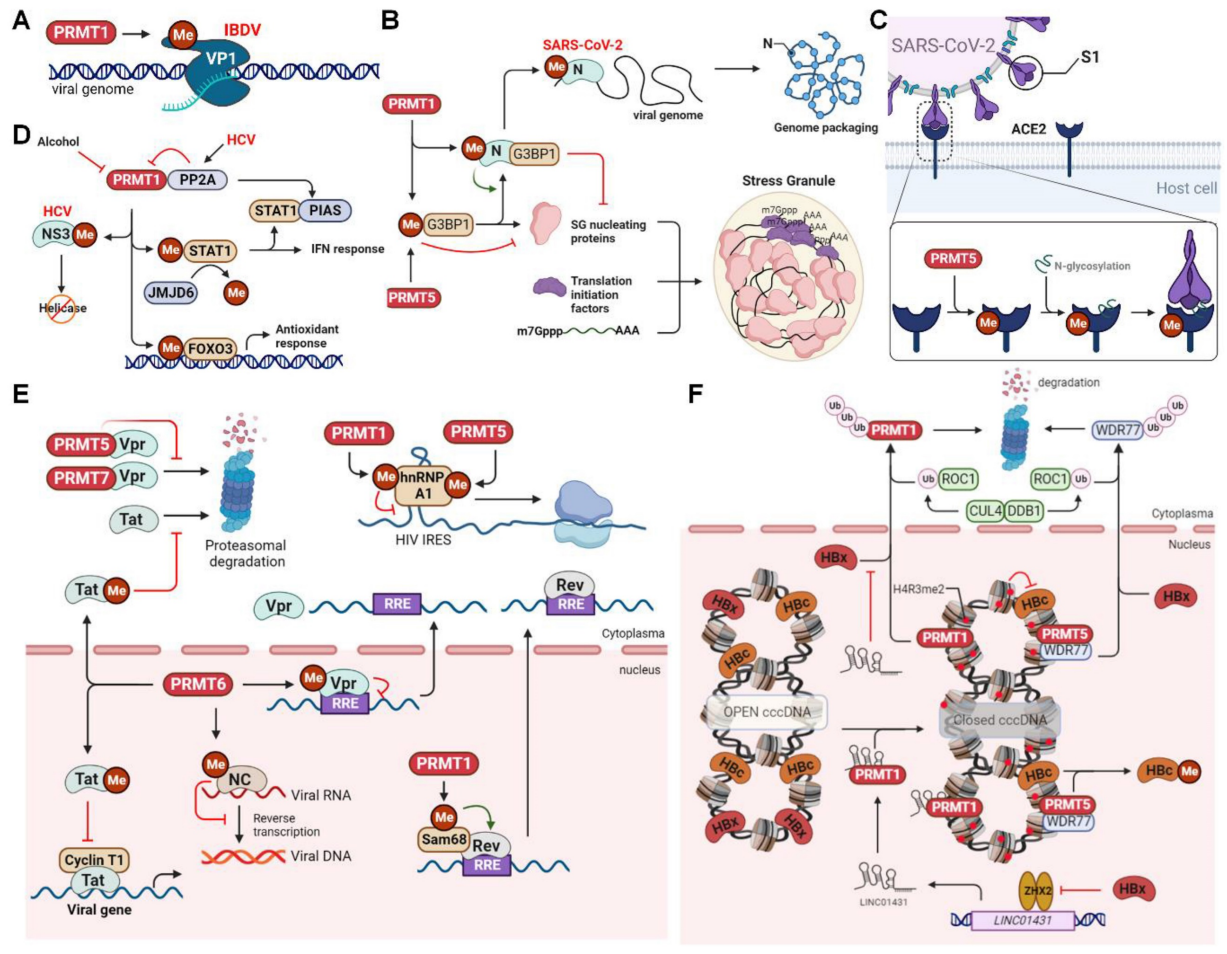
** PRMTs regulate RNA virus infection. (A)** PRMT1 methylates VP1 to promote IBDV replication. **(B)** PRMTs methylate G3BP1 and SARS-CoV-2 N protein to inhibit stress granule formation. PRMT1 also methylates N protein to promote viral genome packaging. **(C)** PRMT5 methylates ACE2 to promote its N-glycosylation and interaction with SARS-CoV-2 S1 protein. **(D)** PRMT1 inhibits HCV NS3 helicase activity and enhances STAT1-mediated interferon response or FOXO3-mediated anti-liver injury response via methylation. PRMT1 activity is also inhibited by PP2A or alcohol. **(E)** PRMT6 methylates HIV Vpr to inhibit nuclear export of viral RRE-containing RNA, or methylates NC to inhibit reverse transcription, or methylates Tat to inhibit its proteasomal degradation and Cyclin T1-mediated viral gene expression. PRMT1 methylates Sam68 to promote viral RNA nuclear transport or methylates hnRNP A1 to inhibit IRES-mediated viral protein translation, the latter of which is enhanced by PRMT5 methyaltion of hnRNP A1. PRMT5 or PRMT7 also interact with Vpr to inhibit its proteasomal degradation. **(F)** PRMT1 and PRMT5 catalyse histone H4R3me2 to inhibit HBV viral transcription. PRMT5 also methylates HBc to promote its dissociation from HBV cccDNA. The viral protein HBx triggers proteasomal degradation of PRMT1 and WDR77 or inhibits the expression of LINC01431, which can interact with and enhance PRMT1 stability by blocking HBx-mediated ubiquitination and degradation. ACE2, Angiotensin‐converting enzyme 2; cccDNA, covalently closed circular DNA; CUL4, cullin 4; DDB1, DNA damage-binding protein 1; FOXO3, forkhead box O3; G3BP1, Ras GTPase-activating protein-binding proteins 1; HBV, hepatitis B virus; HCV, hepatitis C virus; HIV, human immunodeficiency virus; IBDV, infectious bursal disease virus; IRES, internal ribosomal entry site; JMJD6, Jumonji C domain-containing protein 6; PIAS1, protein inhibitor of activated STAT 1; PP2A, protein phosphatase 2A; PRMT, protein arginine methyltransferase; ROC1, regulator of cullins 1; RRE, rev response element; SARS-CoV-2, severe acute respiratory syndrome coronavirus 2; STAT1, signal transducer and activator of transcription 1; WDR77, WD-40 repeat-containing protein77; ZHX2, zinc fingers and homeoboxes 2; Me, methylation; Ub, ubiquitination.

Infectious bursal disease virus (IBDV), a segmented dsRNA virus belonging to the *Birnaviridae* family, infects B lymphocytes in the bursa of Fabricius and causes a highly contagious, acute, and immunosuppressive infectious bursal disease in young chickens. The RNA-dependent RNA polymerase protein VP1 is involved in viral genome replication, transcription, and packaging, and its catalytic activity can be regulated by PTMs, such as sumoylation and phosphorylation 144,145. Arginine methylation by PRMT also affects VP1 activity and viral infection (Figure 7A). IBDV infection recruits PRMT5 to accumulate in the cytoplasm, forming punctate structures with VP1, where PRMT5 methylates VP1 at R426, increasing its polymerase activity, thereby promoting viral replication 146. Additionally, PRMT1 promotes IBDV infection by inhibiting innate antiviral immune response 147. In mammals, innate immunity activated by MAVS is critical in defending against IBDV infection 148. MAVS is activated by two key pattern-recognition receptors (PRRs), retinoic acid-inducible gene-I (RIG-I) and melanoma differentiation-associated protein 5 (MDA5). This activation leads to the recruitment and activation of TANK-binding kinase 1 (TBK1) and interferon regulatory factor 3 (IRF3), ultimately promoting the production of type I interferon (IFN). Consequently, IBDV has developed multiple strategies to evade or suppress host innate immunity, and PRMT1-mediated methylation is an example of one such immune evasion approach 147,149. PRMT1 interacts with the MAVS to prevent the subsequent production of IFN-β and to facilitate efficient viral replication 147. Further studies are required to determine whether PRMT1 targets MAVS via its methylation activity.

### PRMTs in (+)ssRNA virus infection

#### Coronaviridae

The global spread, mutation, and immune escape of severe acute respiratory syndrome coronavirus 2 (SARS-CoV-2), a positive-sense strand RNA virus of the *Coronaviridae* family, have given rise to the COVID-19 pandemic, resulting in substantial loss of life and extensive economic repercussions in recent years. The viral nucleocapsid (N) protein is a conserved RNA-binding protein responsible for RNA synthesis, capsid assembly, and genomic RNA synthesis 150. The N protein also impairs stress granule (SG) formation, an antiviral host defense that induces innate immune responses. It does so by inhibiting viral protein synthesis through sequestration of the SG nucleation factor, G3BP1 151. Notably, cellular phosphatases such as SRPK1 and glycogen synthase kinase 3 can phosphorylate the central RG-rich linker region, affecting the aforementioned functions of the N protein 16,152,153. In addition, arginine methylation by PRMT affects protein-protein and protein-RNA interactions with the N protein, which are necessary for viral production 154. Specifically, PRMT1 methylates the N protein at R95 to suppress G3BP1-mediated SG formation (Figure 7B). The methylation of R95 and R177 is also essential for the binding of the N protein to the viral 5'-UTR RNA, which is a prerequisite for ribonucleoprotein formation and viral genome packaging. Accordingly, inhibition of PRMT1 by MS023 reduces viral replication 154. Furthermore, PRMT5-mediated arginine methylation of angiotensin-converting enzyme 2 (ACE2), the host protein serving as a membrane receptor for the viral spike 1 (S1) protein, at R671 is essential for the interaction between ACE2 and S1 (Figure 7C). Such methylation is believed to enhance N-glycosylation of ACE2, thus promoting its binding to the receptor-binding domain of the S1 protein 155. The PRMT5 inhibitor efficiently attenuates ACE2 binding to S1 and dramatically inhibits infection by SARS‐CoV‐2 and its beta, delta, and omicron variants.

#### Flaviviridae

Chronic hepatitis C virus (HCV) infection can cause inflammatory necrosis of the liver, fibrosis, cirrhosis, and, in severe cases, hepatocellular carcinoma. HCV nonstructural protein 3 (NS3) is a serine protease and RNA helicase involved in viral protein processing and genome unwinding 156. Interestingly, PRMT1 methylates NS3 at R1493 (R467 in the helicase domain) to reduce its RNA-binding ability (Figure 7D) 157. In contrast, HCV upregulates the expression of protein phosphatase 2A (PP2A), which directly binds to PRMT1 and inhibits its methyltransferase activity, thereby increasing the helicase activity of NS3 158. PP2A also inhibits PRMT1-mediated methylation of signal transducer and activator of transcription 1 (STAT1), promoting the binding of unmethylated STAT1 to its inhibitor PIAS and interrupting STAT1-mediated antiviral interferon signaling 159. In addition, HCV upregulates the expression of JMJD6, an arginine demethylase, to reduce the methylation of STAT1 and the resultant antiviral signaling 5. Furthermore, PRMT1 methylates the transcription factor FOXO3 at R248 and R250, enhancing its stability and nuclear translocation during HCV infection (Figure 7D) 160. FOXO3 controls the hepatic antioxidant response and is a protective factor against alcoholic liver injury. Its loss exacerbates liver damage and leads to alcoholic hepatitis 161. Conversely, alcohol inhibits PRMT1 activity and impairs arginine methylation of FOXO3, promoting its nuclear export and cytoplasmic degradation and reducing nuclear transcriptional activity. Therefore, PRMT1-mediated methylation acts as a protective mechanism against the detrimental hepatic consequences of chronic HCV infection.

The West Nile virus (WNV) is a widespread flavivirus of the *Flaviviridae* family, which is transmitted by mosquitoes and causes arboviral encephalitis 162. For viral genome cyclization during WNV RNA synthesis and replication, the nonstructural viral protein NS5 requires assistance from the host factor AU-rich element-binding protein 1 (AUF1) isoform p45 163. Through its RNA chaperone function, AUF1 p45 can destabilize the stem structure at the 3′end of the viral RNA to accelerate structural rearrangement and viral RNA cyclization. Importantly, PRMT1-mediated arginine methylation within the C-terminal RGG motif (R272/278/280/282/345) enhances AUF1 p45 activity, enabling methylated AUF1 p45 to exhibit a higher RNA-binding affinity and more effectively stimulate viral RNA synthesis 164. PRMT1-mediated methylation enhances the RNA chaperone activity of AUF1 p45 to support viral 5'-3' RNA-RNA interactions and accelerate viral RNA cyclization, potentially by altering its secondary structure. Investigating whether arginine methylation is a universal mechanism utilized by other mosquito-borne viruses to facilitate viral replication would be an interesting avenue of research.

#### Hepeviridae

The hepatitis E virus (HEV) belongs to the family* Hepeviridae* and causes acute and chronic hepatitis and can even lead to fatalities worldwide. Various HEV genotypes, including genotypes 1, 2, 3, 4, and 7, have been associated with human infections. HEV ORF1 is a replicase that cooperates with host factors and nonstructural proteins to promote viral genome replication 165. During infection, a replication complex is formed by several host proteins, including KIF11, EIF4B, PRMT5, WDR77, ANKFY1, KCTD5, HSD17B10, STK38, and TAB1, which associate with ORF1 to regulate HEV replication 166. In particular, PRMT5, in cooperation with WDR77, methylates ORF1 at the R458 position to inhibit HEV replication 166. However, it remains unclear whether methylation affects ORF1 replicase activity or its interaction with RNA, thereby restricting viral replication and HEV infection.

### PRMTs in reverse transcribing virus infection

#### Retroviridae (+ssRNA-RT)

Human immunodeficiency virus (HIV) belongs to the *Retroviridae* family, and its productive replication is dramatically affected by the nuclear export of viral RNA, encompassing unspliced, singly spliced, or fully spliced variants. Fully spliced RNA encodes the viral proteins Nef, Tat, and Rev, whereas singly spliced RNA is alternatively spliced to generate viral proteins such as Vpr and Vpu 167. HIV Tat is a transactivator that controls viral gene expression, while Rev is an RNA export factor that binds to the Rev Response Element (RRE) of unspliced and singly spliced HIV RNA, facilitating nuclear-to-cytoplasmic transport. Interestingly, the activity and protein-protein interactions of several viral proteins, such as Vpr, Rev, and Tat, are regulated by PRMTs. For example, PRMT5 and PRMT7 interact with Vpr to prevent proteasomal degradation, thereby enabling viral replication (Figure 7E) 168. In contrast, PRMT6 methylates Vpr within the N-terminal RG motif, diminishing its ability to bind to the RRE, which, in turn, hinders the nuclear export of viral RNA 169. PRMT6 also interacts with Tat, methylating it at R52/R53 and impairing its interaction with cyclin T1 in the transactivation region of viral RNA. Consequently, cyclin T1-dependent Tat transcriptional activation is abolished, resulting in reduced gene expression and, ultimately, HIV production 170,171. However, PRMT6 overexpression in cells with undetectable PRMT6 expression does not affect Tat transactivation, suggesting a cell type-specific inhibitory function for PRMT6 172. In addition, PRMT6-mediated methylation inhibits the nucleolar retention and proteasomal degradation of Tat, which may increase its cytoplasmic and extracellular levels and potentially contribute to viral pathogenesis 173,174. Furthermore, PRMT6 methylates HIV-1 nucleocapsid proteins (NC) at R10 and R32, thereby reducing its efficacy in RNA annealing and viral reverse transcription 175. Importantly, the anti-HIV activity of PRMT6 requires automethylation at R35, which increases its stability and anti-retroviral activity 111.

PRMTs have also been found to regulate several host factors, such as the Src-associated protein of 68 kDa in mitosis (Sam68), heterogeneous nuclear ribonucleoprotein (hnRNP) A1, and viral proteins that influence HIV infection. Sam68, a multifunctional RNA-binding protein, interacts with Rev and directly transports Rev-bound RRE-containing RNA to the nuclear pore complex for nuclear export in a CRM1-independent or -dependent manner 176,177. Sam68 also positively regulates HIV RNA translation in the cytoplasm 178. Notably, Sam68 is methylated by PRMT1 within the RG motif, which is necessary for its nuclear localization and ability to assist in the nuclear export of HIV RNA (Figure 7E) 179. In addition, hnRNP A1 acts as a trans-acting factor at the internal ribosomal entry site (IRES), enhancing IRES activity and promoting the IRES-dependent translation of viral RNA 180,181. HnRNP A1 is also methylated by PRMT1 within its RGG motif *in vivo* and *in vitro*. However, unlike Sam68, such arginine methylation weakens its RNA-binding capability and inhibits its activity as an IRES trans-acting factor 182-184. In contrast, PRMT5 induces symmetrical dimethylation of hnRNP A1 at R218 and R225, enhancing its transcriptional stimulation to upregulate IRES activity and viral protein synthesis (Figure 7E) 185. Finally, CARM1 catalyzes histone H3R26 methylation to prevent the recruitment of the super elongation complex and suppress the long terminal repeat (LTR)-mediated transcription of viral genes 186. Specific inhibition of CARM1 activates viral transcription and disrupts latent HIV-1 infection.

Human T-cell lymphotropic virus type 1 (HTLV-1) is another member of the *Retroviridae* family that causes adult T-cell leukemia. Unlike HIV-1, CARM1 binds to HTLV-1 Tax, a Tat homologue, and enhances transactivation of viral LTR, resulting in histone H3 methylation and viral production 187. Additionally, PRMT5 plays a role similar to that of HIV-1 by methylating hnRNP A1 to promote HTLV-1 IRES activity and viral protein synthesis 188. PRMT5 is also upregulated in HTLV-1-transformed T cells, and its enzymatic activity is essential for cell survival and pathogenesis of HTLV-1-associated diseases 189. Interestingly, PRMT5 plays an opposite role in bovine leukemia virus (BLV) infection, which is closely related to HTLV-1 and causes bovine leukosis and persistent lymphocytosis in cattle. PRMT5 is upregulated during the early stages of BLV infection to restrict viral gene expression, syncytium formation, and glycosylation of the viral gp51 protein. Inhibition of PRMT5 results in enhanced BLV infection 190. Thus, PRMTs play complex roles in antiretroviral therapy.

Foamy virus (FV), a spumavirus belonging to the *Retroviridae* subfamily of* Spumaretrovirinae*, generally causes endemic diseases in nonhuman primates but can occasionally transmit to humans 191. Similar to all retroviruses, the FV Gag protein tethers the pre-integration complex to nuclear chromatin for viral integration and exerts multiple functions throughout the viral life cycle 192. Notably, PRMT1 and PRMT5 interact with and methylate Gag at R540 to regulate its nuclear shuttling, the inhibition of which results in the nucleolar retention of Gag and prevents its binding to chromosomes 193. However, the biological implications of these interactions during viral infections remain unclear.

#### Hepadnaviridae (dsDNA-RT)

Hepatitis B virus (HBV) is a small DNA virus of the *Hepadnaviridae* family whose double-stranded DNA genome forms a covalently closed circular DNA (cccDNA) in the nucleus, which interacts with histones and non-histones to create a minichromosome. This minichromosome serves as a template for viral transcripts and pregenomic RNA (pgRNA). The transcriptional activity of cccDNA is regulated by various histone PTMs, such as H3K4me3 and H3K27ac, and negatively regulated by H3K9me3, H3K27me3, and H4R3me2s 194. In the context of HBV infection, PRMT1 is recruited to cccDNA and catalyzes H4R3me2a modification, which, in turn, alters the acetylation status of cccDNA, ultimately resulting in the suppression of HBV transcription (Figure 7F) 195. However, HBV X protein (HBx), a viral protein, counteracts the inhibitory role of PRMT1 by recruiting several host cellular partners, such as p300/CBP, PACF, and HAT1, to promote histone acetylation and enhance cccDNA transcription and viral replication 196,197. HBx directly inhibits PRMT1′s methyltransferase activity and interfers with CUL4-DDB1-dependent polyubiquitination of PRMT1 to promote HBV replication 198. In addition, HBx regulates the activity of the transcription factor ZHX2 to suppress the transcription of long noncoding RNA (lncRNA) LINC01431, which interacts with and enhances PRMT1 stability by blocking HBx-mediated ubiquitination and degradation. This ultimately results in PRMT1-mediated cccDNA hypoacetylation and transcriptional silencing (Figure 7F) 199.

In addition to PRMT1, PRMT5 restricts HBV replication by epigenetically repressing cccDNA transcription via histone H4R3me2s modification. This process requires the assistance of WDR77, which acts as a non-catalytic component to enhance the substrate-binding affinity and methyltransferase activity of PRMT5 200. Histone methylation by PRMT5 disrupts the interaction between the cccDNA minichromosome and HBV core protein (HBc), as well as the human SWI/SNF chromatin remodeler. This subsequently reduces RNA polymerase II loading on cccDNA. Additionally, PRMT5 directly methylates HBc at residues R150 and R156, which regulates cytoplasm-to-nucleus shuttling and potential ubiquitination. These processes further influence its function in viral genome maturation, pgRNA degradation, and viral particle release 201. In contrast, the viral protein HBx triggers CUL4-DDB1-mediated ROC1 ubiquitin E3 ligase to promote the ubiquitination and proteasomal degradation of WDR77, leading to decreased methyltransferase activity of PRMT5 and enhanced viral replication 202. Furthermore, PRMT5 inhibits the interaction between pgRNA and viral polymerase to impair pgRNA degradation, independent of its methyltransferase activity 200.

Finally, PRMT1 is implicated in hepatitis delta virus (HDV) infection, a satellite virus that requires the helper virus HBV for virus assembly and production. HDV RNA encodes only one protein, the hepatitis delta antigen (HDAg). Its small form, S-HDAg, forms nuclear speckles to regulate RNA replication, while its large form, L-HDAg, is required for viral assembly 203. PRMT1-mediated methylation of S-HDAg at R13 within the RGG motif of the RNA-binding domain is essential for S-HDAg RNA-binding activity and viral RNA replication. In contrast, unmethylated S-HDAg is transported to the cytoplasm, leading to defective RNA replication 204. Further studies are required to investigate how methylated S-HDAgs interact with distinct components of host RNA polymerases to modulate genomic and anti-genomic RNA replication 205.

### PRMTs regulate antiviral innate immunity

**Figure 8:**
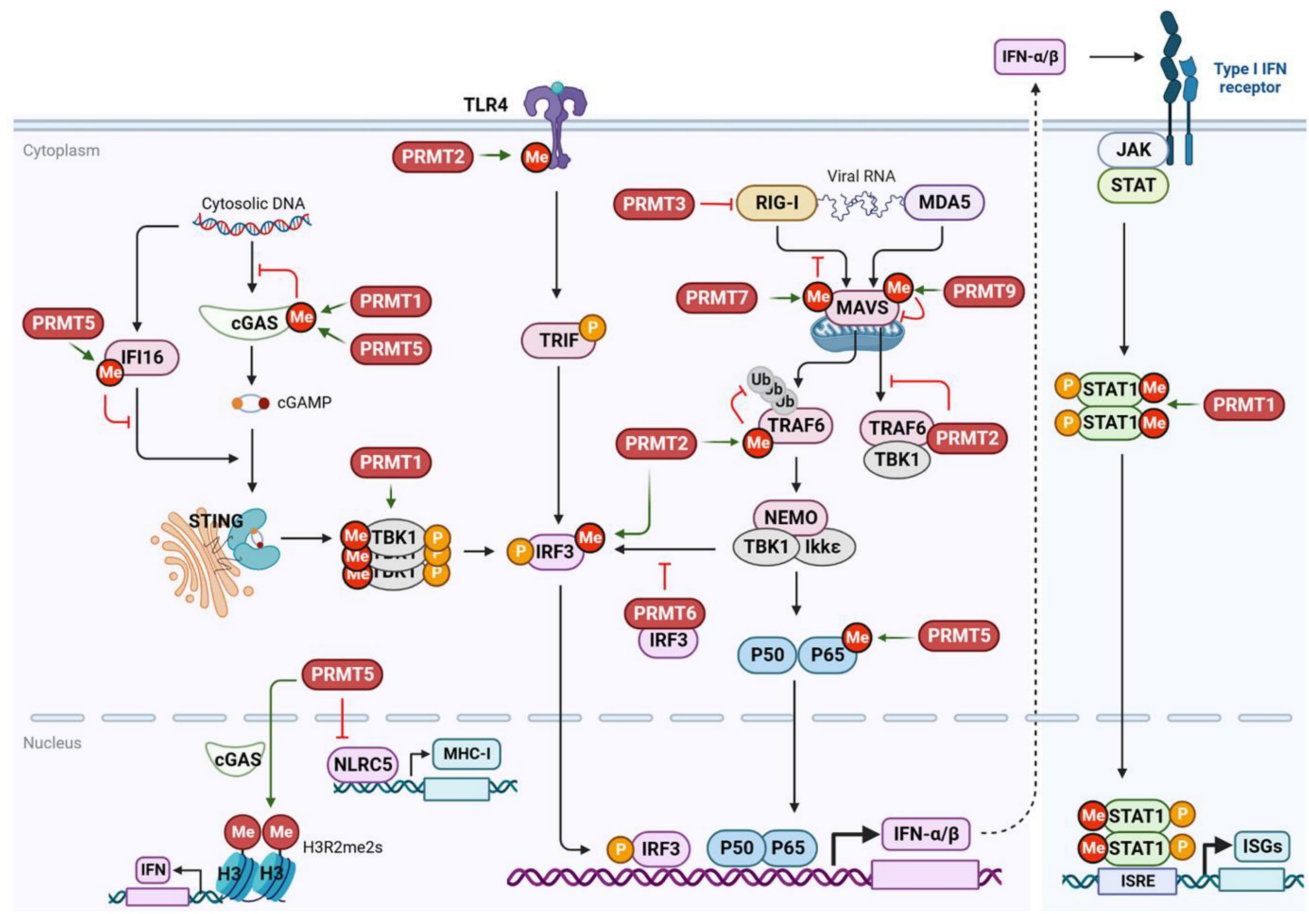
** PRMTs regulate antiviral innate immunity.** PRMT1 methylates cGAS to repress the DNA sensor cGAS/STING signaling pathway, while methylating TBK1 to promote its activation and downstream signals. PRMT1 also methylates STAT1 to promote its nuclear DNA binding and IFN response. PRMT2 stimulates the TLR4/IRF3 pathway to enhance IFN-β production and methylates TRAF6 to prevent its ubiquitination and interaction with MAVS. PRMT3 inhibits RIG-1 activation. PRMT5 methylates cGAS and IFI16 to inhibit cGAS/STING, while interacting with nuclear cGAS to promote H3R2me2s and IFN gene expression. PRMT5 also negatively regulates the transcription of NLRC5, which is required for antigen presentation. PRMT6 sequesters IRF3 to inhibit its activation. PRMT7 methylates MAVS to inhibit its interaction with RIG-I, while PRMT9 methylates MAVS to inhibit its aggregation and autoactivation. cGAS, cyclic GMP-AMP synthase; IFI16, interferon gamma inducible protein 16; IFN, interferon; IRF3, interferon regulatory factor 3; ISG, interferon-stimulated gene; JAK, Janus kinase; MAVS, mitochondrial antiviral signaling protein; MDA5, Melanoma differentiation-associated gene 5; MHC-1, major histocompatibility complex, class I; NEMO, inhibitor of nuclear factor kappa B kinase regulatory subunit gamma; NLRC5, NLR family CARD domain containing 5; RIG-1, retinoic acid-inducible gene I; PRMT, protein arginine methyltransferase; STAT1, signal transducer and activator of transcription 1; STING, stimulator of interferon gene; TBK1, TANK binding kinase 1; TLR4, toll like receptor 4; TRAF6, TNF receptor associated factor 6; TRIF, TIR domain containing adaptor molecule 1; Me, methylation; P, phosphorylation; Ub, ubiquitination.

Increasing evidence suggests that PRMTs catalyze arginine methylation of host cellular proteins involved in antiviral immunity (Figure 8 and Table 4). Different PRRs recognize viral DNA or RNA to activate various transcription factors, such as IRF3, IRF7, and NF-kB, through various downstream signaling cascades, inducing the production of type I IFN and inflammatory factors 206. IFNs further induce the expression of IFN-stimulating factors (ISGs) and many antiviral proteins through the activation of the JAK-STAT signaling pathway to suppress viral infection and stimulate the immune cell-mediated antiviral response.

Cyclic guanosine monophosphate-adenosine monophosphate (cyclic GMP-AMP, cGAMP) synthase (cGAS) is a key DNA recognition receptor that is activated by pathogenic DNA in the cytoplasm. It catalyzes the production of cGAMP, which promotes endoplasmic reticulum-Golgi translocation and dimerization of stimulator of interferon genes (STING) 207. Once activated, STING recruits and phosphorylates the TBK1-IRF3/NF-κB signaling pathway. In addition to its putative involvement in DNA virus sensing, cGAS/STING may also restrict RNA virus infection by recognizing abnormal extranuclear self-DNA released because of genomic DNA damage or mitochondrial stress caused by RNA virus infection 208. Interestingly, upon RNA virus (Sendai virus) and DNA virus (HSV-1) infection, PRMT1 forms oligomers to recruit and methylate the downstream effector TBK1 at R54, R134, and R228, promoting its aggregation and trans-autophosphorylation and the subsequent IRF3-mediated IFN-β production 209. Consistently, *Prmt1* knockout mice show increased susceptibility to viral infections. Additionally, PRMT1 either binds to the intracytoplasmic domain of the IFN receptor or methylates STAT1 at R31, enhancing its DNA-binding activity and transcription factor function, thereby promoting the IFN-stimulated antiviral response 210,211. In the absence of methylation caused by PRMT1 inhibition, phosphorylated STAT1 dimers exhibit an elevated association with the STAT inhibitor PIAS1 (a protein inhibitor of activated STAT 1), resulting in impaired STAT1-DNA-binding and IFN-I response 211. However, in tumor cells, PRMT1 negatively regulates cGAS activation by methylating cGAS at R133 in its N-terminus to prevent dimerization and suppress cGAS/STING signaling 212. Thus, PRMT1 exerts opposite effects on cGAS/STING signaling in different cell types and stressful environments. Higher expression and PRMT1-mediated cGAS methylation may, at least in part, contribute to suppressed basal levels of cGAS/STING signaling in tumors.

In addition to PRMT1, PRMT5 plays a complex regulatory function in the cGAS/STING signaling pathway (Figure 8). During DNA viral infection, PRMT5 negatively regulates the cGAS-mediated antiviral immune response by methylating cGAS at R124 within the RGG/RG motifs to block its DNA-binding activity 213. However, in the case of RNA virus infections (e.g., VZV, SeV, Zika, and NDV), nuclear cGAS recruits and interacts with PRMT5 to demethylate histone arginine (H3R2me2s) of the IFN-β and IFN-α4 promoters. This facilitates chromatin accessibility of IRF3, ultimately boosting IFN-I expression 214. The non-canonical anti-RNA viral function of cGAS is independent of its nucleotidyltransferase and DNA-binding activities, suggesting that cGAS acts as an antiviral cytosolic DNA sensor (DNA virus) or co-transcription factor (RNA virus). The conflicting roles of PRMT5 in cytoplasmic and nuclear cGAS may ensure the accurate duration and strength of IFN-I production. PRMT5 appears to interact with cytoplasmic cGAS, limiting its DNA-binding ability, thereby preventing abnormal inflammation caused by cytosolic self-DNA during stress 215. Upon viral infection, PRMT5 dissociates from cytoplasmic cGAS and moves to the nucleus, where it directly activates cGAS/STING to mobilize antiviral actions (DNA viruses) or interacts with nuclear cGAS to prime innate immune responses (RNA viruses) 214. In addition, PRMT5-mediated symmetric arginine methylation is required for PRR-dependent IFN production in undifferentiated monocytes and activated subsets of T lymphocytes 216. PRMT5 conservatively controls IFN transcription by activating TBK1, c-Jun, and ATF2. PRMT5 also promotes IFN signaling by enhancing the phosphorylation of STAT1 in T cells during the pathogenesis of acute graft-versus-host disease 217. However, the controversial role of PRMT5 in IFN-I production has also been demonstrated. PRMT5 represses the cGAS/STING pathway by methylating IFN-γ-inducible protein 16 (IFI16), another cellular DNA sensor that stimulates STING, at R124. This ultimately attenuates dsDNA-mediated activation of the TBK1-IRF3 signaling in melanoma tumors 218. PRMT5 also transcriptionally inhibits the NLR family CARD domain-containing 5 (NLRC5) to regulate the antigen presentation pathway of MHC class I 218. Thus, PRMT5 exerts paradoxical but rational physiological functions in diverse biological processes and in cells of distinct origins.

Following RNA virus infection, RNA recognition receptors, such as RIG-I, MDA5, and Toll-like receptors (TLRs), rapidly detect and bind viral RNA in the cytoplasm or endosomal compartments to initiate downstream signaling cascades. This initiation of downstream signaling cascades converges upon the activation of the TBK1-IRF3/7 and IKK-NF-kB axes, leading to IFN induction 148,206. RIG-I and MDA5 transmit signals through MAVS, which is localized in the outer mitochondrial membrane. Meanwhile, TLRs recruit either TIR domain-containing adaptor inducing IFN-β (TRIF) adaptor protein or myeloid differentiation primary response 88 (MyD88) to transmit signals from viral infections. In response to an RNA viral infection, MAVS is activated to form prion-like aggregates via K63-linked polyubiquitination, which is mediated by the ubiquitin E3 ligase TRIM31 219. MAVS activity is also tightly regulated by PTMs to fine-tune IFN-I production, as inappropriate, persistent, or spontaneous aggregation of MAVS leads to systemic autoimmunity 220. PRMT9 has recently been identified as a negative MAVS regulator that methylates the R41 and R43 residues to prevent their aggregation and auto-activation during resting conditions (Figure 8) 221. Upon viral infection, PRMT9 dissociates from the mitochondria, leading to MAVS activation and IFN-I stimulation. In addition, PRMT7 monomethylates MAVS at R52 to attenuate its binding to TRIM31 and subsequent TRIM31-mediated polyubiquitination, as well as its interaction with RIG-I. This results in the suppression of MAVS aggregation and activation of the RLR signaling pathway 114. Conversely, viral infection-induced automethylation of PRMT7 at R32 alleviates the suppression of MAVS activation and promotes its interaction with the MAVS-recruited ubiquitin E3 ligase SMURF1, leading to K48-linked polyubiquitination and proteasomal degradation of PRMT7 114. Thus, decreased MAVS methylation facilitates aggregate formation and activation of the innate antiviral response.

Furthermore, following viral infection, PRMT6 interacts with and sequesters IRF3 independently of its methyltransferase activity. Consequently, the interaction between TBK1 and TRF3 is disrupted, leading to the suppression of IRF3-mediated IFN-I production 222. In zebrafish, PRMT3 interacts with RIG-I to inhibit its activation and subsequent IRF3 phosphorylation, thereby negatively regulating antiviral responses to spring viremia of the carp virus (SVCV) and grass carp reovirus (GCRV) infection 223. Additionally, PRMT2 binds and methylates TRAF6 at R100 within the C-terminus, preventing its K63-linked auto-ubiquitination and activation of the downstream TBK1/IKKε signaling pathway 224. The N-terminal of PRMT2 also competes with MAVS for TRAF6 binding and disrupts the recruitment of TBK1/IKKε to MAVS. Thus, PRMT6 has a bidirectional impact, negatively regulating antiviral innate immunity against SVCV and GCRV infections.

Apart from viral infection, PRMT-mediated arginine methylation also enhances IFN-β production in the TLR4/IRF3 signaling pathway 225. Following LPS stimulation, PRMT1-3 have been shown to methylate TLR4 and IRF3, with PRMT2 having the most prominent impact (Figure 8). PRMT2 can either methylate TLR4 at R731 and R812, or IRF3 at R285, and methylated TLR4 at R812 directly binds to methylated IRF3. IRF3 methylation also induces its dimerization and promotes nuclear import, resulting in increased IFN-β production 225. Furthermore, the methylation of TLR4 by PRMT1 or PRMT2 increases IRF3 transcriptional activity, irrespective of LPS stimulation. Further investigation is needed to determine whether PRMT2-mediated TLR4 methylation augments antiviral innate responses via other downstream effectors, such as the MyD88-dependent signaling pathway.

Finally, several PRMTs positively regulate NF-kB signaling and target gene expression in response to different stresses. For instance, CARM1 acts as a promoter-specific transcriptional co-activator of NF-kB that interacts with p300 and the NF-kB subunit p65, augmenting the recruitment of NF-kB to the chromatin of specific genes 226. PRMT1 forms a complex with poly (ADP-ribose) polymerase 1 and directly interacts with the NF-kB subunit p65 to co-activate NF-kB-dependent gene expression synergistically with CARM1 227. In addition, upon TNF-α stimulation, PRMT6 promotes p65 nuclear shuttling and interacts with p65 at selective NF-κB target promoters as a co-activator, enhancing gene expression 228. Furthermore, PRMT5 directly interacts with and dimethylates p65 at R30, thereby promoting gene expression 229. Given that the methyltransferase activity of PRMT1 and CARM1 is essential for their co-activator function, further research is required to investigate whether PRMT1 and CARM1, similar to PRMT5, directly methylate p65 to activate NF-κB. In contrast, PRMT2 inhibits NF-κB-dependent transcription by preventing the nuclear export of IκB-α. This leads to the nuclear accumulation of IκB-α, which curbs NF-κB DNA- binding and the expression of genes targeted by NF-κB 230. However, it remains to be determined whether PRMTs-mediated regulation of NF-κB has a potential role in the antiviral innate immune response.

### PRMTs as emerging antiviral targets

Given the pivotal roles of PRMTs in viral infections and antiviral innate immune responses, PRMTs are emerging as potential therapeutic antiviral targets. However, *in vitro* studies have identified a limited number of PRMT inhibitors as potent antiviral agents. This could be attributed to the lack of specific PRMT inhibitors available for exploring the virus-associated functions of PRMTs. For instance, a type I PRMT inhibitor (MS0233) and PRMT5-specific inhibitor (GSK3326595) have been shown to inhibit SARS-CoV-2 replication 154. IBDV replication is also impaired by the PRMT5 inhibitor HLCL-61 146. General PRMT inhibitors (AdOx and AdoHcy) efficiently inhibit HSV-1 replication 124. In zebrafish, the PRMT3 inhibitor SGC707 has been found to effectively inhibit SVCV and GCRV infections 223. Furthermore, the CARM1 specific inhibitor 7 g (1-benzyl-3,5-bis-(3-bromo-4-hydroxy-benzylidene) piperidin-4-one) effectively reverses HIV latency and facilitates reactivation 186. This suggests that 7 g may serve as a novel latency-disrupting compound and presents a possible combinatorial strategy for eliminating the latent HIV provirus reservoir. To date, several potent PRMT inhibitors with different structural scaffolds have been developed for cancer therapy. Some of these inhibitors, such as the PRMT1 inhibitor GSK3368715 and PRMT5 inhibitors PF-06939999, GSK3326595, and JNJ-64619178, have been investigated in clinical trials 231. Additional studies are required to examine the antiviral properties of these compounds.

## Conclusions

Overall, the accumulating evidence highlights the complex role of protein arginine methylation in viral infections and antiviral innate immunity. This sheds light on a new avenue for understanding the pathogenesis of viral diseases and offers potential opportunities for antiviral therapy. Protein arginine methylation, governed by PRMTs, affects a diverse array of host cellular proteins and various functional viral proteins, including viral capsids (e.g., SARS-CoV-2 N and HBV HBc), mRNA exporters (e.g., HSV ICP27 and HCMV pUL69), transcription factors (e.g., ADV L4-100K and HIV Tat), and latency regulators (e.g., EBV EBNA1 and KSHV LANA). This methylation affects their activity, subcellular localization, protein-protein or protein-nucleic acid interactions, and participation in various stages of the viral life cycle, such as genome replication, RNA nuclear export, alternative splicing, transcription, translation, immune evasion, and the switch governing viral latency reactivation. However, the regulatory mechanisms of arginine methylation in viral infections are not fully understood, owing to the pleiotropic roles of PRMTs on diverse substrates and crosstalk with other PRMT proteins that scavenge or share substrates. For example, the coordination of arginine methylation complexities remains unclear, as some PRMTs constitutively methylate a wide range of substrates, while others are inducible in response to stimuli and PTMs. In addition, the transcriptional and post-transcriptional regulation of PRMT enzymes remains relatively unexplored, with only a limited number of protein partners and PTMs known to regulate their activity, stability, and function. The application of methylation proteomics holds promise for unveiling additional interactive links between viral proteins and PRMTs. A comprehensive understanding of the functionally diverse substrates of PRMTs could offer more profound insights into the regulation of arginine methylation in viral infection and pathogenesis. Notably, PRMT8 and PRMT9, as well as their hosts and viral substrates, require further investigation. The exploitation of tissue-specific viruses may leverage their distinct tissues and subcellular distributions. Furthermore, the identification of critical arginine demethylases that enable complete methylation and demethylation cycles will further elucidate virus-host interactions and expand the scope of regulatory mechanisms.

Another exciting frontier lies in the development of selective and potent PRMT inhibitors with diverse clinical applications. This could help unravel the downstream biological functions of PRMTs and offer alternative therapeutic options for infectious diseases. However, individual PRMTs may have either positive or negative effects on viral infection, and the broad substrate spectrum of PRMTs suggests that inhibiting PRMTs may have a much broader impact than initially assumed. For instance, PRMT1 and PRMT5 have been shown to play pro-oncogenic roles, and their corresponding inhibitors are currently at the forefront of clinical investigations 2,13. In the case of oncogenic viruses (e.g., HBV), the application of PRMT inhibitors may be complicated because of the inhibitory effects of PRMTs on HBV infection 195,200,201. Further research is needed to differentiate the effects of PRMT inhibitors on virus-infected cells versus immune cells, as the integrative antagonism provided by immune cells is likely to determine the therapeutic efficacy. In addition, novel interference strategies such as proteolysis-targeting chimeras may provide an alternative route for PRMT modulation 232. Collectively, the recognition of the regulation of arginine methylation provides a crucial foundation for addressing diseases associated with viral infections.

## Figures and Tables

**Table 1:** Histone arginine methylation by PRMTs.

PRMT	Substrate	Modification	Biological function
PRMT1	H2A	H2AR3me2a	Transcriptional activation
		H2AR11me2a	/
	H4	H4R3me2a	Transcriptional activation
PRMT2	H3	H3R8me2a	Transcriptional activation
PRMT3	H4	H4R3me2a	Transcriptional activation
CARM1	H3	H3R2me2a	Transcriptional repression
		H3R17me2a	Transcriptional activation
		H3R26me2a	Transcriptional activation
		H3R42me2a	Transcriptional activation
PRMT5	H2A	H2AR3me1/me2s	Transcriptional repression
	H3	H3R2me1/me2s	Transcriptional activation
		H3R8me2s	Transcriptional repression/ activation
	H4	H4R3me2s	Transcriptional repression/ activation
PRMT6	H2A	H2AR3me2a	Transcriptional activation
		H2AR11me2a	/
		H2AR29me2a	Transcriptional repression
	H3	H3R2me2a	Transcriptional repression
		H3R42me2a	Transcriptional activation
	H4	H4R3me2a	Transcriptional activation
PRMT7	H2A	H2AR3me1	Transcriptional repression
	H2B	H2BR29me1	/
		H2BR31me1	/
		H2BR33me1	/
	H3	H3R2me1/me2s	Transcriptional activation
	H4	H4R3me1	Transcriptional repression
		H4R17me1	/
		H4R19me1	/
PRMT8	H4	H4R3me2a	Transcriptional activation

**Table 2:** Activity regulation of PRMTs by PTMs.

PRMT	Regulator	PTM	Amino acid	Biological effect
PRMT1	CDK5	Phosphorylation	S307	Promotes its lysosomal translocation to methylate WDR24, activating the mTORC1 pathway and tumor growth
	DNA-PK	Phosphorylation	/	Promotes H4R3me2s methylation and senescence-associated secretory phenotype
	CSNK1a1	Phosphorylation	S248, T285, S289	Controls its genomic targeting and suppresses GRHL3-mediated terminal differentiation
	ULK3	Phosphorylation	/	Promotes H4R3me2s and keratinocyte self-renewal and tumorigenesis
	/	Phosphorylation	Y291	Inhibits its methyltransferase activity and substrate binding
	TRIM48	Ubiquitination (K48)	/	Proteasomal degradation
	USP11	Deubiquitination	/	Enhances its interaction with MRE11 and DNA damage repair
PRMT3	/	Phosphorylation	Y87	Essential for its methyltransferase activity and RNA maturation
CARM1	PKA	Phosphorylation	S448	Promotes its binding to ERα
	PKC	Phosphorylation	S228	inhibits its methyltransferase activity and promotes its binding to ERα
	GSK3B	Phosphorylation	T132	Protect it from ubiquitin-mediated proteasomal degradation
	SKP2	Ubiquitination	K471	Proteasomal degradation
	CARM1	Auto-Methylation	R550	Affects ERα-mediated transcription and pre-mRNA splicing
	OGT	O-GlcNAcylation	S595, S598, T601, T603	Affects substrate specificity
PRMT5	TRAF6	Ubiquitination (K63)	K85, K95, K200, K227, K240, K241	Enhances its methyltransferase activity and substrate binding
	CHIP	Ubiquitination (K48)	K240, K241, K248, K259, K275, K302, K329, K333, K343, K354, K380, K387	Proteasomal degradation
	JAK2K539L (*JAK2 exon12 mutation*)	Phosphorylation	Y297, Y304, Y307	Disrupts its interaction with MEP50 and inhibits its methyltransferase activity in myeloproliferative disease
	PKCι	Phosphorylation	S15	Activates NF-κB and promotes tumor growth
	Akt	Phosphorylation	T634	Modulates a 14-3-3/PDZ interaction switch
	SGK	Phosphorylation	T634	Modulates a 14-3-3/PDZ interaction switch
	LKB1	Phosphorylation	T132, T139, T144	Promotes its methyltransferase activity and interaction with substrates (e.g., MEP50, pICln, RiOK1)
	Src	Phosphorylation	Y324	Interferes with AdoMet binding, suppressing its methyltransferase activity and triggering DNA damage-related cell death
	ULK3	Phosphorylation	/	Promotes H4R3me2s and keratinocyte self-renewal and tumorigenesis
	ROK	Phosphorylation	T80	Promotes H4R3me2s to trigger proto-oncogene activation and tumor formation
	MP	De-phosphorylation	T80	Inhibits H4R3me2s to inhibit proto-oncogene activation and tumor formation
	CARM1	Methylation	K562	Essential for its methyltransferase activity to repress human γ-globin expression via H4R3me2s
PRMT6	PRMT6	Auto-Methylation	R35	Essential for its stability and antiretroviral activity
	CK2α	Phosphorylation	S11, T21	Inhibits its ubiquitin-dependent proteasomal degradation, promoting RCC1 methylation and self-renewal and tumorigenicity of glioma
PRMT7	PRMT7	Auto-Methylation	R531	Promotes E-cadherin expression and breast cancer progression via H4R3me1
	PRMT7	Auto-Methylation	R32	Relieves its suppressive function on MAVS activation and antiviral innate immunity
PRMT8	PRMT8	Auto-Methylation	R58, R73	Blocks SAM binding to inhibit its enzymatic activity

**Table 3:** PRMTs regulate viral infection.

Virus family	Virus	PRMT	Viral targets	Role	Biological effect
*dsDNA*					
*Herpesviridae*	HSV	PRMT1	ICP27	Positive	Methylates ICP27 to promote its nuclear export and viral replication
	HCMV	PRMT6	pUL69	Positive	Methylates pUL69 to promote viral mRNA export and replication
	EBV	PRMT1	EBNA1	Positive	Methylates EBNA1 to promote its nuclear location; Methylates NCL to suppress EBNA1 expression and promote viral immune evasion
		PRMT3	/	Positive	Methylates NCL to suppress EBNA1 expression and promote viral immune evasion;
		CARM1	/	/	Upregulated by virus infection
		PRMT5	EBNA1	Positive	Methylates EBNA1 to promote its nuclear location and viral gene expression
			EBNA2	Positive	Enhances EBNA2-mediated transcription
	KSHV	PRMT1	LANA	Negative	Methylates LANA to modulate viral latent activation
		PRMT5	ORF59	Negative	ORF59 interacts with and suppresses PRMT5-mediated H4R3 methylation to promote viral reactivation
*Papillomaviridae*	HPV	PRMT1	E6	Negative	E6 interacts with and suppresses PRMT1-mediated histone methylation of p53-responsive gene to favor viral pathogenesis
		CARM1	E6	Negative	E6 induces CARM1 degradation and suppresses p53 methylation to favor viral pathogenesis
*Adenoviridae*	ADV	PRMT1	L4-100K	Positive	Methylates L4-100K to ensure viral late protein synthesis and virus yield
*dsRNA*					
*Birnaviridae*	IBDV	PRMT1	/	Positive	Promotes viral replication via suppressing IFN-β production
		PRMT5	VP1	Positive	Methylates VP1 to facilitate viral replication
*Reoviridae*	GCRV	PRMT3	/	Positive	Promotes viral replication via binding to RIG-I and suppressing IRF3 phosphorylation
		PRMT6	/	Positive	Promotes viral replication via suppressing TBK1-IRF3/7 signaling
		PRMT7	/	Positive	Promotes viral replication via suppressing RIG-I signaling
(+)ssRNA					
*Coronaviridae*	SARS-CoV-2	PRMT1	N	Positive	Methylates N protein to suppress stress granule formation and promote viral replication
		PRMT5	/	Positive	Methylates ACE2 to promote viral infection
*Flaviviridae*	HCV	PRMT1	NS3	Negative	Methylates NS3 to suppress its helicase activity and viral replication, which is reversed by PP2A
	WNV	PRMT1	/	Positive	Methylates AUF1 p45 to promote viral RNA synthesis
*Hepeviridae*	HEV	PRMT5	ORF1	Negative	Methylates ORF1 to inhibit viral replication
(-)ssRNA					
*Rhabdoviridae*	SVCV	PRMT3	/	Positive	Promotes viral replication via binding to RIG-I and suppressing IRF3 phosphorylation
		PRMT6	/	Positive	Promotes viral replication via suppressing TBK1-IRF3/7 signaling
		PRMT7	/	Positive	Promotes viral replication via suppressing RIG-I signaling
ssRNA-RT					
*Retroviridae*	FV	PRMT1	Gag	/	Regulates the nucleolar location of Gag
		PRMT5	Gag	/	Regulates the nucleolar location of Gag
	HIV	PRMT1	/	Positive	Methylates Sam68 to promote the export of unspliced HIV RNAs
		CARM1	/	Negative	Represses LTR-mediated transcription via H3R326me2a;
		PRMT5	/	Positive	Methylates hnRNP A1 to promote viral protein synthesis
			Vpr	Positive	Prevents Vpr degradation to supports viral replication
		PRMT6	Tat	Negative	Methylates and inhibits Tat nucleolar retention, transcriptional activity and interaction with TAR/Cyclin T1 to inhibit viral replication
			NC	Negative	Methylates and inhibits NC to restrict viral RNA annealing and reverse transcription
			Rev	Negative	Methylates and inhibits Rev-mediated viral RNA export
		PRMT7	Vpr	Positive	Prevents Vpr degradation to supports viral replication
	HTLV-1	CARM1	Tax	Positive	Enhances Tax transactivation and histone H3 methylation
		PRMT5	/	Positive	Methylates hnRNP A1 to promote viral protein synthesis;
	BLV	PRMT5	/	Positive	Regulates viral gene expression and syncytium formation
dsDNA-RT					
*Hepadnaviridae*	HBV	PRMT1	HBx	Negative	Represses viral cccDNA transcription via H4R3me2a; Degraded by HBx-mediated ubiquitination
		PRMT5	HBc	Negative	Interacts with HBc and represses viral cccDNA transcription via H4R3me2s; Inhibits HBV core particle DNA production; Regulates HBc cytoplasmic-nuclear transport
			HBV P	Negative	Prevents pregenomic RNA encapsidation via interfering the interaction between HBV P and RNA
Defective virus					
*Deltavirus*	HDV	PRMT1	S-HDAg	Positive	Methylates S-HDAg to promote viral replication

**Table 4:** PRMTs regulate antiviral immunity.

PRMT	Targets	Role	Molecular mechanism
PRMT1	TBK1	Positive	Methylates TBK1 at R54/R134/R228, promoting its aggregation and trans-autophosphorylation
	STAT1	Positive	Methylates STAT1 at R31, enhancing its DNA binding activity and interferon-stimulated response
	cGAS	Negative	Methylates cGAS at R133, preventing its dimerization and suppress the cGAS/STING signaling
PRMT2	TLR4/IRF3	Positive	Methylates TLR4 at Arg731/Arg812 or IRF3 at Arg285 to enhance IFN-β production
PRMT2 (*Zebrafish*)	TRAF6	Negative	Methlyates TRAF6 at R100 inhibits its autoubiquitination and MAVS-mediated TBK1/IKKε activation
PRMT3 (*Zebrafish*)	/	Negative	Interacts with RIG-I and suppresses IRF3 phosphorylation
PRMT5	cGAS	Negative	Methylates cGAS at Arg124, blocking its DNA binding activity
	cGAS (nuclear)	Positive	Interacts with nuclear cGAS to promote interferon expression via H3R2me2s
	IFI16	Negative	Methylates IFI16 at Arg124, attenuating dsDNA-mediated activation of TBK1-IRF3 signaling
	NLRC5	Negative	Suppresses NLRC5 expression to regulate antigen presentation
PRMT6	IRF3	Negative	Sequesters IRF3 and blocks TBK1-IRF3 signaling
PRMT7	MAVS	Negative	Catalyzes MAVS monomethylation at Arg52 to inhibit its interaction with TRIM31 and RIG-I
	/	Negative	Epigenetically suppresses RIG-I/MDA5 pathway via H4R3me2s
PRMT7 (*Zebrafish*)	/	Negative	Suppresses RLR pathway
PRMT9	MAVS	Negative	Catalyzes MAVS methylation at Arg41/Arg43 to inhibit its aggregation and autoactivation

## Abbreviations

ACE2: angiotensin-converting enzyme 2
ADMA: asymmetric dimethyl arginine
ASK1: apoptosis signal-regulating kinase 1
AUF1: AU-rich element binding protein 1
BLV: bovine leukemia virus
CARM1: coactivator-associated arginine methyltransferase 1
cccDNA: covalently closed circular DNA
CDK5: cyclin-dependent kinase 5
cGAMP: cyclic guanosine monophosphate-adenosine monophosphate
cGAS: cyclic guanosine monophosphate-adenosine monophosphate synthase
CHIP: carboxy-terminus of Hsc70 interacting protein
CK2α: casein kinase 2α
COPR5: cooperator of protein arginine methyltransferase 5
CSNK1a1: casein kinase 1α
DDB1: DNA damage-binding protein 1
DNA-PK: DNA-dependent protein kinase
EBNA: Epstein-Barr nuclear antigen
EBV: Epstein-Barr virus
ERα: estrogen receptor α
E6P: E6-associated protein
FOXO3: forkhead box O3
FV: foamy virus
GCRV: grass carp reovirus
GRHL3: grainyhead like transcription factor 3
GSK-3β: glycogen synthase kinase 3β
G3BP1: ras GTPase-activating protein-binding proteins 1
HBV: hepatitis B virus
HCMV: human cytomegalovirus
HCV: hepatitis C virus
HDAg: hepatitis delta antigen
HEV: hepatitis E virus
HIV: human immunodeficiency virus
hnRNP A1: heterogeneous nuclear ribonucleoprotein A1
HPV: human papillomavirus
HTLV-1: human T-cell lymphotropic virus type 1
HSV: herpes simplex virus type 1
IBDV: infectious bursal disease virus
IFI16: interferon gamma inducible protein 16
IFN: interferon
IRF3: interferon regulatory factor 3
IRES: internal ribosomal entry site
ISG: interferon-stimulated gene
JAK: janus kinase
JMJD6: Jumonji C domain-containing protein 6
KSHV: kaposi sarcoma-associated herpesvirus
LANA: latency-associated nuclear antigen
LKB1: liver kinase B1
LTR: long terminal repeat
MAVS: mitochondrial antiviral signaling protein
MDA5: Melanoma differentiation-associated gene 5
MEP50: methylosome protein 50
MHC-1: major histocompatibility complex, class I
MMA: monomethyl arginine
MP: myosin phosphatase
MRE11: meiotic recombination 11
MyD88: myeloid differentiation primary response 88
NCL: nucleolin
NEMO: inhibitor of nuclear factor kappa B kinase regulatory subunit gamma
NF-κB: nuclear factor kappa B
NLRC5: NLR family CARD domain containing 5
NS3: nonstructural protein 3
PDZ: proteins containing PDZ domain
pgRNA: pregenomic RNA
PIAS1: protein inhibitor of activated STAT 1
pICln: chloride nucleotide-sensitive channel 1A
PKA: protein kinase A
PKC: protein kinase C
PKCι: Protein kinase C iota
PRMT: protein arginine methyltransferase
PP2A: protein phosphatase 2A
PRR: pattern-recognition receptors
PTM: post-translational modifications
RBP: RNA-binding proteins
RCC1: ribosomal chromatin condensation 1
RG: arginine-glycine
RIG-1: retinoic acid-inducible gene I
RioK1: Rio kinase 1
ROC1: regulator of cullins 1
ROK: RhoA activating kinase
RRE: rev response element
SAM: S-adenosyl-L-methionine
SARS-CoV-2: severe acute respiratory syndrome coronavirus 2
SDMA: symmetric dimethyl arginine
SG: stress granule
SGK: serum and glucocorticoid inducible protein kinase
SKP2: S-phase kinase-associated protein 2
SR: splicing factors
SRPK1: serine-arginine protein kinase 1
STAT1: signal transducer and activator of transcription 1
STING: stimulator of interferon gene
SVCV: spring viraemia of carp virus
TBK1: TANK binding kinase 1
TLR4: toll like receptor 4
TPL: tripartite leader sequence
TRIF: TIR domain containing adaptor molecule 1
TRIM48: tripartite motif 48
TRAF6: TNF receptor associated factor 6
Trx: thioredoxin
ULK3: unc51-likekinase 3
USP11: ubiquitin specific peptidase 1
WDR: WD-40 repeat-containing protein
WNV: west nile virus
ZHX2: Zinc Fingers And Homeoboxes 2
100K: late region 4 (L4) 100-kDa protein
53BP1: p53-binding protein 1
